# Comparative Coastal Risk Index (CCRI): A multidisciplinary risk index for Latin America and the Caribbean

**DOI:** 10.1371/journal.pone.0187011

**Published:** 2017-11-02

**Authors:** Juliano Calil, Borja G. Reguero, Ana R. Zamora, Iñigo J. Losada, Fernando J. Méndez

**Affiliations:** 1 Center for the Blue Economy, Middlebury Institute of International Studies, Monterey, California, United States of America; 2 Institute of Marine Sciences, University of California Santa Cruz, Santa Cruz, California, United States of America; 3 The Nature Conservancy, Santa Cruz, California, United States of America; 4 Universidad de Cantabria, Santander, Cantabria, Spain; 5 Environmental Hydraulics Institute “IH Cantabria”, Universidad de Cantabria, Santander, Cantabria, Spain; Universidade de Vigo, SPAIN

## Abstract

As the world’s population grows to a projected 11.2 billion by 2100, the number of people living in low-lying areas exposed to coastal hazards is projected to increase. Critical infrastructure and valuable assets continue to be placed in vulnerable areas, and in recent years, millions of people have been displaced by natural hazards. Impacts from coastal hazards depend on the number of people, value of assets, and presence of critical resources in harm’s way. Risks related to natural hazards are determined by a complex interaction between physical hazards, the vulnerability of a society or social-ecological system and its exposure to such hazards. Moreover, these risks are amplified by challenging socioeconomic dynamics, including poorly planned urban development, income inequality, and poverty. This study employs a combination of machine learning clustering techniques (Self Organizing Maps and K-Means) and a spatial index, to assess coastal risks in Latin America and the Caribbean (LAC) on a comparative scale. The proposed method meets multiple objectives, including the identification of hotspots and key drivers of coastal risk, and the ability to process large-volume multidimensional and multivariate datasets, effectively reducing sixteen variables related to coastal hazards, geographic exposure, and socioeconomic vulnerability, into a single index. Our results demonstrate that in LAC, more than 500,000 people live in areas where coastal hazards, exposure (of people, assets and ecosystems) and poverty converge, creating the ideal conditions for a perfect storm. Hotspot locations of coastal risk, identified by the proposed Comparative Coastal Risk Index (CCRI), contain more than 300,00 people and include: El Oro, Ecuador; Sinaloa, Mexico; Usulutan, El Salvador; and Chiapas, Mexico. Our results provide important insights into potential adaptation alternatives that could reduce the impacts of future hazards. Effective adaptation options must not only focus on developing coastal defenses, but also on improving practices and policies related to urban development, agricultural land use, and conservation, as well as ameliorating socioeconomic conditions.

## Introduction

Backing away from estimates from less than a decade ago, the United Nations now predicts that the world population is unlikely to stabilize by the end of the century. The global population, currently at 7.46 billion, is increasing by nearly 230,000 people every day, at a growth rate of 1.18% per year [1]. In the next 15 years, the global population is expected to grow by an additional 1 billion, reaching 11.2 billion people by 2100 [1]. Concurrently, the number of people living in low elevation coastal areas, exposed to natural hazards, continues to increase [2]. There is a clear trend of coastal populations growing globally, with an estimated 230% increase (from 2000 to 2030) in the size of urban areas within the Low Elevation Coastal Zone (LECZ)—defined as “the contiguous area along the coast that is less than 10 meters above sea level”, and which accounts for only 2% of the planet’s total land area [3,4]. Moreover, critical infrastructure and valuable assets continue to be placed in areas exposed to coastal hazards [5].

In 2013, almost 22 million people were displaced by extreme weather events across the globe, with 37 events displacing at least 100,000 people each [5]. All but one of the top 15 largest displacements were related to typhoons or floods, with at least three million people displaced from coastal areas [6]. In 2012, more than 30 million people were displaced worldwide by disasters related to climate and weather events [6]. From 1995 to 2015, worldwide losses resulting from minor but recurrent natural hazards, including flash floods, landslides, and storms, reached $94 billion [7].

Natural events are not the only reason why disasters occur. Disaster risk is defined by a complex interaction between physical hazards and the vulnerability of a society or social-ecological system, and its exposure to such hazards [8]. The disaster risk-poverty nexus has been well documented [8–12]; Poor communities, which have limited or no access to insurance, are usually less resilient to disasters and suffer a disproportionate share of losses resulting from such events [9]. Furthermore, the occurrence of disasters reduces income and consumption levels, further aggravating poverty [9].

Social, political and economic conditions are often ignored determining factors for the consequences of the onset of disasters [13–16]. Coastal risks are amplified by challenging socioeconomic dynamics, including poorly planned urban development, income inequality, and poverty [17]. Lack of access to critical resources including food, fresh water, shelter, medicine and evacuation routes, can greatly intensify the damaging effects of coastal hazards [16]. Finally, income inequality is frequently associated with larger damage [18]. Inequality increases poverty and creates processes of social and political exclusion, possibly resulting in social instability, reduced accountability and enabling corruption [18].

There is a well-documented need for studies that explicitly integrate exposure and vulnerability to coastal hazards, disaster risk management, and adaptation [19,20]. Further, multidisciplinary approaches are an effective way to evaluate and solve complex environmental and social problems [20–24].

Previous research addressed the exposure of critical resources to coastal hazards in Latin America and the Caribbean (LAC). In 2011, the Economic Commission for Latin America and the Caribbean (ECLAC) published an assessment of the risks and impacts of climate change in coastal areas of LAC [25]. The 2011 study produced a comprehensive high-resolution database containing more than 15,000 coastal segments of roughly 5km by 10km length each. Individual coastal segments contain multiple attributes related to natural hazards (e.g. significant wave height, storm surge, and wind) and geographic exposure (e.g. urban and cropland area, beaches, and ecosystems, critical infrastructure, and Gross Domestic Product (GDP)). Coastal risks and hotspots were evaluated for flooding and coastal erosion, resulting from both sea-level rise and extreme weather events. Losada et al. (2013) [26] studied historical sea-level rise and extreme sea levels in LAC, while Reguero et al. (2013) [27], and Izaguirre et al. (2012) [28] described changes in wave conditions in the region. More recently, Reguero et al. (2015b) [29] assessed the exposure of people, land, and built capital to coastal flooding in LAC under current and future conditions of sea level rise (SLR), El Niño-induced SLR, and storms.

While the studies above address important knowledge needs, they do not incorporate important drivers of risk, such as poverty and inequality. Building on the works from ECLAC (2011) [25], Losada et al. (2013) [26], Reguero et al. (2015a, 2015b) [29,30], and based on the methods developed by Camus et al. (2011) [31], and Ramos et al. (2012) [32], we present a method that identifies critical drivers of coastal risks and isolates hotspots of coastal vulnerability. The aim of this study is to develop a Comparative Coastal Risk Index (CCRI) by combining multiple variables related to the three dimensions of risk (coastal hazards, geographic exposure, and socioeconomic vulnerability) into a single value.

### Study region—Latin America and the Caribbean

The total population in the LAC region in 2014 was approximately 623 million people (with an annual growth rate of 1.1% between 2010 and 2015) [33]. The GDP in LAC in 2014 was approximately $5.7 trillion. Brazil, Mexico and Argentina had the highest GDPs in 2014 ($2.4 trillion, $1.2 trillion and $0.5 trillion, respectively) [34]. In Colombia, Venezuela, Costa Rica, El Salvador, and Panama, more than 30% of the total population is located in the LECZ [35]. In 2000, roughly 32.2 million people lived in the LECZ in LAC [36,37].

This study focuses on coastal areas (i.e. under 10m in elevation and within 5km of the coast) of LAC, and includes more than 13,000 unique coastal segments covering more than 59,000km of coastline. The study area includes a total population of almost 23 million people in 26 countries, and is bounded by Mexico (north and west), Chile (south), and Brazil (east).

Despite recent advances in promoting economic and social development, efforts in LAC have failed to significantly reduce poverty [38]. There is a large number of people in the region with no access to basic services including water and sanitation [38], a situation that greatly increases the vulnerability of coastal populations to natural hazards.

From 1972 to 2010, 88 natural disasters caused nearly 310,000 deaths and 236 billion dollars in damages (2015 dollars) in LAC [39]. During the same period, 63 meteorological events caused roughly $118 billion in damages [39]. Storms and hurricanes were responsible for 40 disasters, resulting in 50.2% of all deaths, and almost 40% of total damages [39]; in 1998, a single event, Hurricane Mitch, caused more than 23,000 deaths in Central America. From 1972 to 2010, El Niño and La Niña events caused 17 disasters in the region, resulting in approximately 50% of all damages and 4.1% of all deaths [39].

## Methods and materials

### a) Methodology

This study employs a combination of machine learning clustering techniques (Self Organizing Maps (SOM) and K-Means) and a spatial index, to classify and rank coastal areas according to coastal risk. Hazards, exposure, and vulnerability data were combined to calculate a comparative coastal risk index (CCRI).

The benefits of clustering the data before applying an index are multiple. The categorization of multivariate datasets according to similar attributes simplifies the analysis; SOM provide intuitive visualization of results, greatly facilitating the analysis of multivariate sets in a two dimensional plane. Further, the clustering analysis produces risk profiles across the region allowing areas of similar risk profiles to be easily identified; the most relevant variables of hazards, exposure, and vulnerability can be traced back from the final risk index, to individual clusters. Finally, clustering techniques allow the analysis of large datasets at a low computational cost.

The benefits of the proposed approach include: (i) the identification of hotspots and key drivers of coastal risk; (ii) the ability to process large-volume multidimensional and multivariate datasets, effectively reducing sixteen variables related to coastal hazards, geographic exposure, and socioeconomic vulnerability, into a single index; and (iii), clustering of coastal areas according to similar attributes, where consistent risk reduction strategies may be applied to minimize future risk.

**Fig 1:**
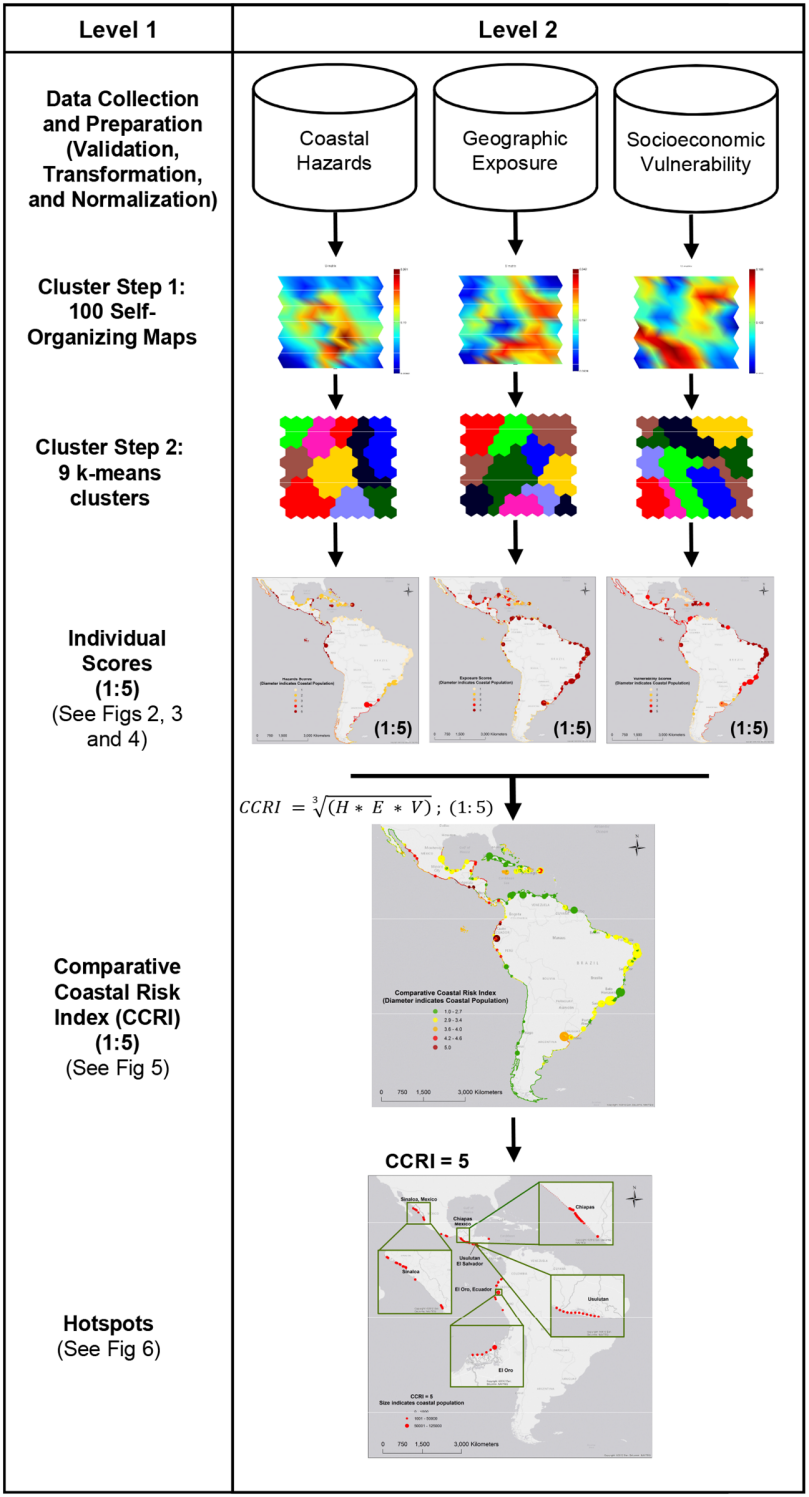
Methods flow chart.

An overview of the three methodological steps is shown in Fig 1. First, variables within each risk dimension (hazards, exposure, and vulnerability) were clustered. Second, individual scores for each risk dimension were calculated. Finally, a Comparative Risk Index (CCRI) was calculated.

Within the context of this study, risk is defined as the geometric mean between the individual scores describing various degrees of *hazards*, *exposure*, *and vulnerability*. Areas with higher individual scores receive a higher comparative risk index value. For this first implementation of CCRI, an equal weights approach was chosen. However, future applications may consider different weighting scenarios.

### b) Data

The methodology leverages several datasets, including the ones published by Reguero et al. (2013 and 2015a, 2015b) [27,29], Losada et al. (2013) [26], Izaguirre et al. (2013) [40], and ECLAC (2011) [41]. Several new attributes were appended to the original datasets, including: cumulated cyclone winds (used as a proxy for hurricanes), GDP, Gini coefficient of inequality, and Infant Mortality Rates (IMR). A description of the individual variables used in each score follows:

#### Coastal hazards

Coastal hazards may be related to extreme weather events (e.g. storm surge and winds from tropical storms), or to higher-frequency, low intensity events (e.g. sea level rise due to El Niño events) [16,42,43]. Table 1 contains a description of the coastal hazards variables included in the study.

**Table 1:** Coastal hazards variables.

Coastal Hazards Score Components	Data Source	Resolution (degrees of Latitude or km)	Period of Data	Unit
**Wave Energy**	Reguero et al. (2015a) [30]	0.25° (Caribbean) 0.50° remaining areas	1948–2008	W/m^2^
**Storm Surge 99% (m)**	Losada et al. (2013) [26]	0.25° (Caribbean) 0.50° remaining areas	1948–2010	m
**El Nino 1997–1998 (m)**	Losada et al. (2013) [26]	0.50°	1997–1998	m
**Significant Wave Height Ratio (HS 12 / HS mean)**	Reguero et al. (2013 and 2015b) [27,29]	0.25° (Caribbean) 0.50° remaining areas	1948–2008	ratio
**Cumulated Tropical Cyclone Winds**	Global Risk Data Platform, United Nations Environment Programme (UNEP), [44]	2km	1975–2007	km (km/h*h)

#### Geographic exposure

Geographic exposure is defined as the presence (of people, ecosystems, infrastructure, and assets) in places that could be adversely affected by physical hazards [20]. The variables included in this study representing exposure are: coastal population, GDP, urban area, cropland and various ecosystems.

An ecosystems category was included to reflect the valuable (and often overlooked) services that ecosystems provide. As an example, wetlands and mangroves provide valuable services to neighboring communities in the form of coastal protection, enhancement of fisheries, water filtration, sediment trapping, and many others [45]. As ecosystems are impacted by the onset of coastal hazards, however, their value to coastal communities is diminished, hence their inclusion in the exposure category.

The following ecosystems were included in this study: beaches, mangroves, estuaries, marshes, grasslands, deciduous, mixed and conifer forests, and deserts [26]. Ecosystems data were summarized into three components: (i) beach area; (ii) wetlands (sum of saltmarshes and estuaries); and (iii), coastal forests (sum of mangroves, grasslands, deciduous, and mixed forests).

Table 2 contains a description of the geographic exposure variables included in the study.

**Table 2:** Geographic exposure variables.

Exposure Score Components	Data Source	Resolution	Date	Unit / Year
**Coastal Population**	Reguero et al. (2015b) [29]	1km^2^	2000	Number of People
**% Urban Coverage**	ECLAC (2011) [46]	5km	2000?	Ratio
**% Crop Coverage**	ECLAC (2011) [46]	5km	2011	Ratio
**Beach area**	ECLAC (2011) [46]	5km	2011	Km (km^2^/km)
**Coastal Forests area**	ECLAC (2011) [46]	5km	2011	Km (km^2^/km)
**Wetlands area**	ECLAC (2011) [46]	5km	2011	Km (km^2^/km)
**Per Capita GDP (average)**	Global Risk Data Platform, UNEP [44] (see S2 Appendix for additional sources)	30 arc second resolution, roughly 1 km^2^	2000	USD (year 2000, extrapolated to 2010)

#### Socioeconomic vulnerability

Within the context of this study, socioeconomic vulnerability is described in terms of the ability of a coastal community to cope with and adapt to a coastal hazard that may impact livelihoods and well-being [47]. Poverty and welfare are common indicators of socioeconomic vulnerability, and can be evaluated by proxy variables [48,49]. Vulnerability variables used in this study, namely: Infant Mortality Rate (IMR), Child Malnutrition, GDP, and Income Inequality, are commonly accepted indicators of socioeconomic vulnerability and poverty [48–50]. Table 3 contains a list of the socioeconomic vulnerability variables included in the study.

**Table 3:** Socioeconomic vulnerability variables.

Vulnerability Variable	Data Sources	Resolution	Period	Unit
**Gini coefficient**	The Standardized World Income Inequality Database [51] (see S2 Appendix for additional sources)	National	1995–2012	N/A
**Child Malnutrition Rate (%)**	SEDAC [52] (see S2 Appendix for other sources)	Subnational	1990–2000	%
**Infant Mortality Rate (%)**	SEDAC Center for International Earth Science Information Network (CIESIN) [53] (see S2 Appendix for additional sources)	Subnational	2000	number of deaths
**Per Capita GDP (average)**	Global Risk Data Platform, (UNEP) [44] (see S2 Appendix for additional sources)	Subnational	2010	USD

### c) Clustering analysis

Clustering techniques were used to investigate how coastal areas in the LAC region may be grouped according to similar characteristics of hazards, exposure, and vulnerability. We follow the techniques and recommendations from Camus et al. (2011, and 2016) [31,54], Ramos et al. (2012) [32], and Rueda et al. (2017) [55]. Clustering, in this context, means partitioning of each dataset into smaller groups of similar characteristics. Two clustering algorithms were applied in two subsequent steps. First, the Self-Organizing Maps (SOM) algorithm was applied to distribute 13,426 study units into 100 maps. Second, the K-Means algorithm was applied to further group the 100 resulting SOM maps into 9 clusters (S1 Appendix). The application of two abstraction levels proved more consistent than applying either one independently, confirming the findings from Vesanto and Alhoniemi (2000) [56]. Recent studies applied similar methods of data classification in various research fields [31,57–60].

One of the limitations of the used algorithms is that they do not propose an optimal number of clusters. Therefore, it was necessary to test multiple values for each abstraction level (SOM and K-Means), until it was possible to validate that the resulting clusters indeed consisted of locations with similar features. Several attempts were made to adjust the best number of final clusters (from 2 to 25), with 9 clusters best representing the data. As an index is introduced in the analysis, the issue of the number of clusters is greatly minimized, as similar clusters receive similar values for the final index.

#### Data transformation and normalization

The exposure dataset contains a significant number of coastal segments with no population or GDP (values equal 0), resulting in a highly-skewed distribution. To improve the distribution, the Box-Cox transformation (Eq 1) was applied to the relevant variables.

$${x^{'}}_{\lambda }=\frac{\left(x^{\lambda }-1\right)}{\lambda }$$ (1)

Subsequently, all variables were then transformed to range from 0 to 1. This step ensures that all variables have equal weight in the clustering analysis.

#### Maximum dissimilarity initialization

The Maximum Dissimilarity algorithm was used to pre-select the most distinct values within the dataset as the initial centers for each cluster, ensuring that the resulting clusters are as diverse as possible. [31,61].

#### First clustering step–Self-Organizing Maps (SOM)

The SOM algorithm [62] facilitates the visualization of high-dimensional data by converting nonlinear statistical relationships between multiple dimensions into simple geometric shapes, usually a simple grid of nodes. SOM compresses information but retains the most important relationships of the original data elements. Formally, SOM is the nonlinear mapping of high-dimensional input data into a linear array. Each map unit produced represents a vector, comprised of as many columns as the original dataset (i.e. each hazards, exposure, and vulnerability attributes). The most commonly used SOM output is a topological representation of the data, where each cell represents a cluster and contains a number of data entries, or samples, associated with it. All samples within a cell are similar, and also similar to samples in adjacent cells, while samples assigned to distant cells are less similar. A probability matrix is also produced, which represents the number of records belonging to each cluster. SOM reduced the dimensionality of each data set into a single value for each cluster.

Hazards, exposure, and vulnerability data were clustered independently. Several attempts were made to perform a single cluster analysis utilizing a single database containing all variables. However, clustering results, based on 16 variables, proved to be too complex and cumbersome to be analyzed.

#### Second clustering step–K-Means algorithm

As a second clustering step, the K-Means algorithm was applied to further reduce the 100 SOM groups (resulting from the first clustering step) into 9 clusters. This step was repeated for each category (hazards, exposure, and vulnerability), independently. Using K-means to assign each one of the 100 SOM groups into 9 clusters provides additional benefits. It provides a smaller number of clusters, and the ability to compare similar values that were placed further apart in the SOM maps. K-means cluster centers (mean values) represent prototypes for the records belonging to it. Individual SOM groups were assigned to the prototypes with the closest mean value in a two-step iterative way. On each step, the algorithm calculates a mean value for each cluster, based on the values of the points belonging to it. In a second step, individual points are reassigned to the cluster with the closest value [63]. Values within clusters are more similar, and closer in value to the cluster’s mean value, than other clusters. This process is repeated until points no longer jump between clusters. In this study, the process was repeated 100 times. Several numbers of K-Means clusters were evaluated (from 3 to 25) with the best results achieved with 9 clusters.

#### Hazards, exposure, and vulnerability scores

The next step in the analysis was to assign individual scores to the 9 clusters (for each category), and rank them by intensity. Scores were calculated according the equations described below, and variables within each score were equally weighted.

The rationale behind developing these scores is that areas where more than one variable is present receive a higher score than areas where only one, or no variables are present (e.g. areas impacted by both high wave energy and tropical storm winds would be ranked higher than areas only affected by one of these hazards).

Once scores were calculated, each one of the 9 clusters within each category (hazards, exposure, and vulnerability), was ranked from 1 to 5 (with 1 representing the lowest severity, and 5 representing the highest severity).

One limitation of this approach is the assumption that that the impacts from individual hazards are equal in severity. As an example, we assume that elevated sea levels during an El Niño event have the same impact as areas of high accumulated winds. This assumption is adequate for the objectives and spatial scale of the current analysis, and illustrates a basic implementation of the proposed approach. However, it may not be adequate for other applications of this method, or the selection of local risk reduction strategies. Moreover, the proposed index is not meant to represent a definite result, but rather a starting point, showcasing the benefits of the method presented here. However, the model is flexible, and different weights can be easily assigned to specific variables within the model, making it suitable to be used by diverse stakeholders. Future applications of this model should ensure that each variable receives the appropriate weight that represents the study’s objectives.

#### Coastal hazards score

An overall coastal hazards score was calculated by summing up the values of individual hazard variables at each coastal segment (Eq 2). Variables included in the hazards score are: waves (average between significant weight height ratio, and wave energy), storm surge, wind, and El Niño. Hazards scores for the 9 clusters were ranked from 1 to 5 (low to high), according to severity.

CoastalHazardsScore(HS)=Waves+StormSurge+Wind+ElNiño (2)

#### Geographic exposure score

An overall Exposure Score was calculated by summing up the values of individual exposure variables for each coastal segment. Variables included in the exposure score are: coastal population, GDP, cropland ratio, urban ratio, and coastal ecosystems. Exposure scores for the 9 clusters were ranked from 1 to 5 (low to high) according to severity (Eq 3).

GeographicExposureScore(ES)=CoastalPopulation+GDP+CroplandRatio+UrbanRatio+Ecosystems (3)

Ecosystems data were summarized into three components: (i) beach area; (ii) wetlands (sum of saltmarshes and estuaries); and (iii), coastal forests (sum of mangroves, grasslands, deciduous, and mixed forests). First, linear densities were calculated for each attribute by dividing the total areal coverage of the respective attribute (e.g. total area of coastal forests in the segment, in km^2^) by the length of each segment (in km). As an example, the linear density of forests was calculated by dividing the total area of coastal forests in each coastal segment, by the length of the respective segment. Second, the linear densities of beaches, coastal forests and wetlands were averaged into a broader ecosystems category, calculated for each coastal segment (Eq 4).

Ecosystems=(BeachLinearDensity+CoastalForestsLinearDensity+WetlandsLinearDensity)/3 (4)

#### Socioeconomic vulnerability score

An overall vulnerability score was calculated by summing up the values of individual vulnerability variables for each coastal segment. The following variables were included in the vulnerability score: a social welfare function (SWF), IMR, and malnutrition (Eq 5). Vulnerability score scores for the 9 clusters were ranked from 1 to 5 (low to high) according to severity.

A social welfare function (SWF), which combines GDP and the Gini coefficient was used in the vulnerability score, as it better describes aggregated income and its distribution [64,65] (Eq 6). Areas with higher value of SWF are wealthier, therefor less vulnerable than areas with low values of SWF. Given the inverse relationship between SWF and socioeconomic vulnerability, SWF is negative.

SocioeconomicVulnerabilityScore(VS)=IMR+Malnutrition+SWF (5)

SWF=-(GDP*(1-Giniindex)) (6)

### Comparative Coastal Risk Index (CCRI)

Finally, the CCRI was calculated as the geometric mean of the hazards, exposure, and vulnerability scores, for each coastal segment. CCRI values range from 1 to 5 (Eq 7).

$$Comparative\;Coastal\;Risk\;Index\;(CCRI)=\sqrt[{3}]{\left(HS*ES*VS\right)}$$ (7)

## Results and discussion

The results from this first implementation of CCRI in LAC, which are based on an equal weights approach, are meant to illustrate different kinds of analysis that this method can support. Values described as “high”, “large”, or “low” and “small” are relative to values from other areas in the study region. Nevertheless, they may still be considered higher or lower when compared with other regions of the planet not included in the study.

The following section describes the resulting clusters for each category of CCRI. The 13,426 original coastal segments were reduced to 9 hazards clusters (H1 to H9), 9 exposure clusters (E1 to E9), and 9 vulnerability clusters (V1 to V9).

### Coastal hazards–Clusters and scores

First, coastal hazards data were clustered according to characteristics of: waves, storm surge, wind, and El Niño induced sea level changes. The waves component, was calculated as the average of Significant Wave Height and Wave Energy variables, after they were scaled from 0 to 1. Second, hazards scores were calculated (Eq 2), and range from 1 to 5, with 5 being the most severe. Clusters H1 and H8 received the maximum hazards score, and are characterized by El Niño induced sea levels, and strong cumulated winds, respectively (Table 4).

**Table 4:** Coastal hazards clusters (sorted by hazards score).

Cluster	% of Coastal Segments	Coastal Length (km)	Coastal Population	Most Relevant Attribute	Top 3 Affected Countries (by population)	Hazards Score
**H1**	16%	10,312	2.2 million	Strong El Niño	Mexico, Ecuador, Peru	5
**H8**	7%	4,673	1.0 million	Strong winds	Puerto Rico, Mexico, and Caribbean	5
**H3**	7%	3,628	3.3 million	High Waves	Argentina, Uruguay, Brazil	4
**H4**	8%	4,985	0.6 million	Moderate El Niño	Peru, Puerto Rico and the Dominican Republic	3
**H6**	11%	5,184	27,000	Moderate Waves	Chile and Mexico	3
**H5**	9%	4,147	3.4 million	Weak Storm Surge	Brazil, Argentina, and Chile	2
**H7**	10%	6,213	2.1 million	Moderate winds and weak El Niño	Cuba, Dominican Republic, and Haiti	2
**H9**	8%	5,043	2.0 million	Small waves and Weak El Niño	Mexico, Cuba and Haiti	2
**H2**	24%	15,119	8.3 million	Weak El Niño	Brazil, Venezuela, Colombia	1

As previously discussed, one of the benefits of applying clustering techniques, prior to the index calculation, is that drivers of coastal risks can be traced back to individual clusters for each category (hazards, exposure, and vulnerability).

**Fig 2:**
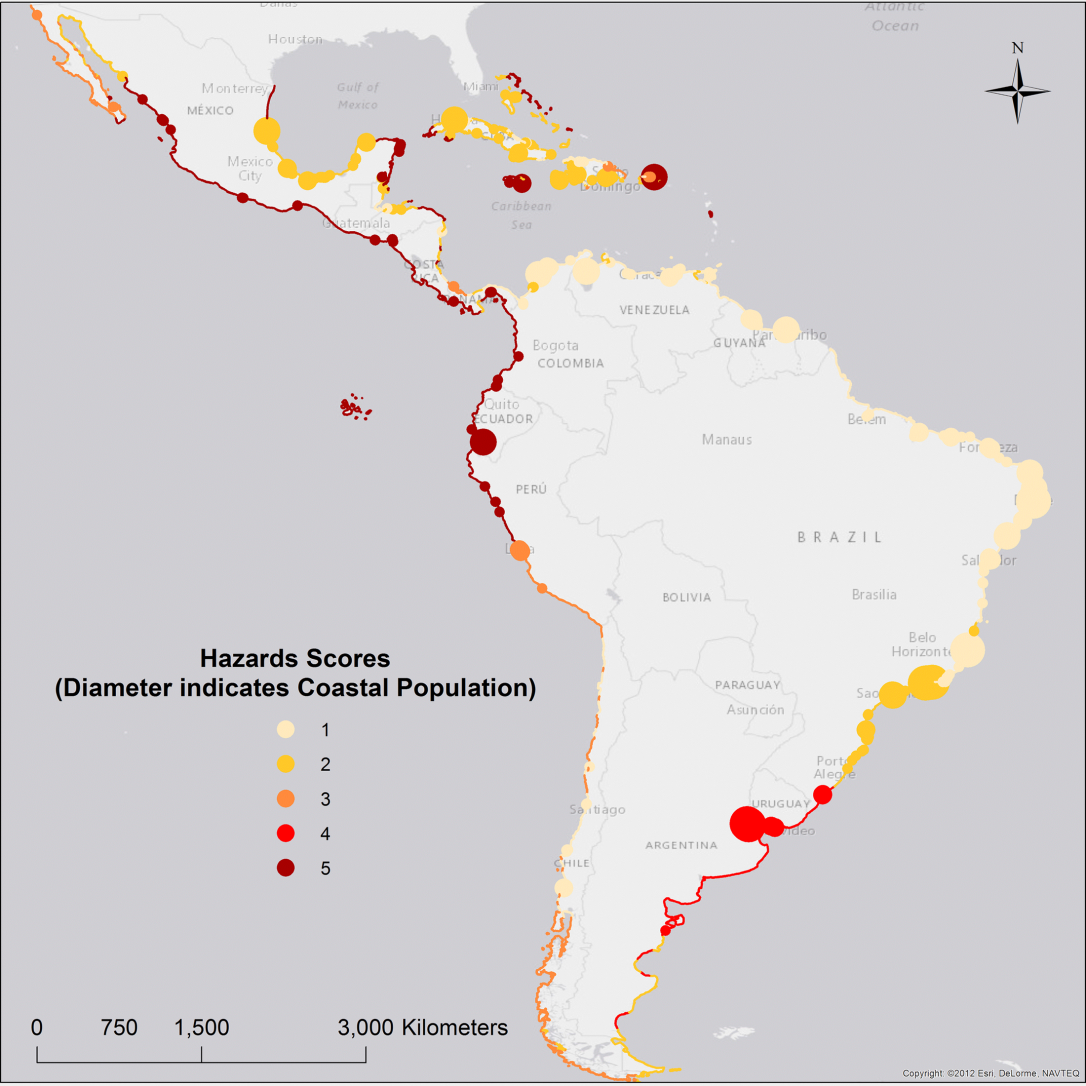
Coastal hazards scores.

The spatial distribution of the hazards clusters (Fig 2) is consistent with recent studies of coastal hazards in the LAC region [26,27,41].

### Geographic exposure–Clusters and scores

**Fig 3:**
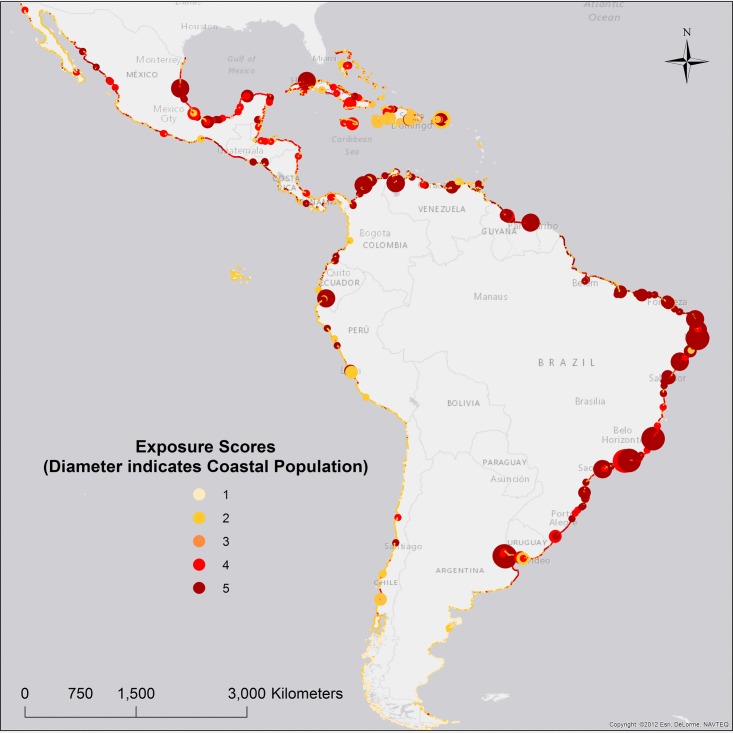
Exposure scores.

First, geographic exposure data were clustered according to characteristics of: coastal population, GDP, urbanization, cropland, and ecosystems. Second, exposure scores were calculated (Eq 3), and range from 1 to 5, with 5 indicating the highest geographic exposure levels. Geographic exposure clusters are not as geographically concentrated as the coastal hazards discussed above. Clusters E1, E8, and E6 received the maximum exposure score. These three clusters include coastal segments with large population. However, E1 includes large urban areas while E8 and E6 are characterized by a more significant presence of ecosystems and croplands, respectively (Table 5, and Fig 3).

**Table 5:** Geographic exposure clusters (sorted by exposure score).

Cluster	% of Coastal Segments	Coastal Length (km)	Coastal Population	Most Relevant Attribute	Top 3 Affected Countries (by population)	Exposure Score
E1	2%	1,410 km	8.0 million	Largest population, GDP, and urban areas	Brazil, Argentina, Mexico	5
E8	4%	2,706 km	2.9 million	Large population, ecosystems and croplands	Brazil, Colombia, and Ecuador	5
E6	7%	4,818 km	2.3 million	Large population and ecosystems	Mexico, Brazil, and Guyana	5
E4	16%	9,821 km	6.1 million	Large population and croplands	Brazil, Mexico, Cuba	4
E7	13%	8,247 km	1.6 million	Croplands and moderate GDP	Haiti, Dominican Republic, and Brazil	3
E9	25%	15,220 km	1.5 million	Moderate GPD	Brazil, Mexico, and Chile	2
E5	7%	4,477 km	0.5 million	Moderate GDP; ecosystems	Peru, Venezuela, and Colombia	2
E3	5%	2,685 km	0	Moderate croplands and GDP	Trinidad Tobago, Mexico, and Haiti	2
E2	20%	9,921 km	1,780	Low presence of all variables	Cuba, Mexico and Belize	1

### Socioeconomic vulnerability–Clusters and scores

**Fig 4:**
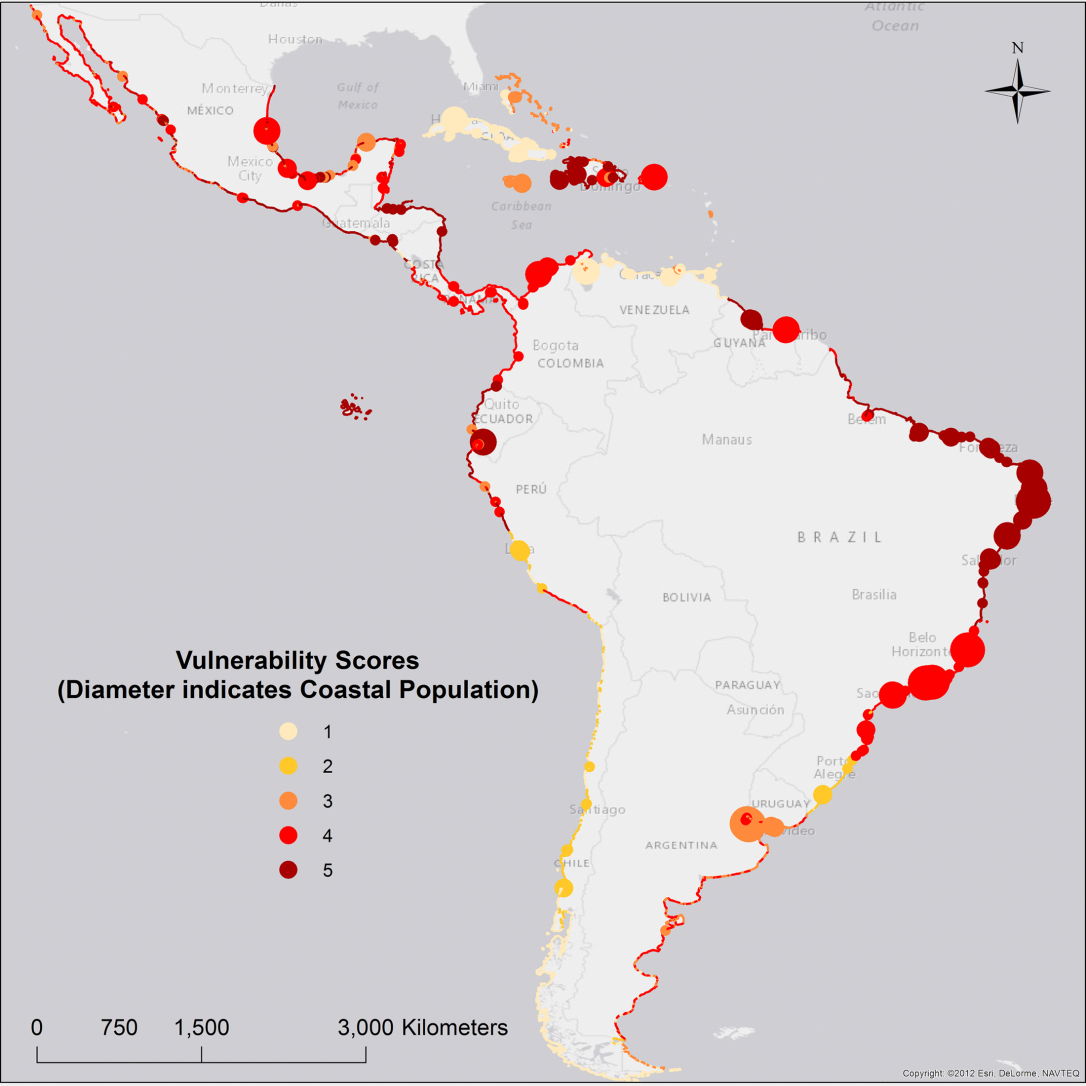
Socioeconomic vulnerability scores.

First, socioeconomic vulnerability data were clustered according to characteristics of: GDP, Inequality, IMR, and malnutrition. Second, vulnerability scores were calculated as the sum of SWF, IMR, and malnutrition (Eq 4), and range from 1 to 5, with 5 indicating the highest socioeconomic vulnerability. Clusters V1, V9 received the maximum vulnerability score, with high IRM, malnutrition, and low social welfare (Table 6, and Fig 4).

**Table 6:** Socioeconomic vulnerability clusters (sorted by vulnerability score).

Cluster	% of Coastal Segments	Coastal Length (km)	Coastal Population	Most Relevant Attributes	Top Countries Affected (by population)	Vulnerability Score
**V1**	10%	6,491	5 million	Highest IMR and malnutrition; low SWF	Haiti, Brazil, and Honduras	5
**V9**	13%	8,324	1.6 million	High IMR and malnutrition; low SWF	Mexico, Guyana, and Ecuador	5
**V3**	14%	8,784	7.7 million	High IMR and malnutrition; low SWF	Brazil, Mexico, and Colombia	4
**V7**	15%	8,567	521,000	High malnutrition; medium IMR; low SWF	Mexico, Argentina and Peru	4
**V8**	2%	1,285	84,000	Medium malnutrition and IMR; low SWF	Brazil, Colombia, and Panama	4
**V4**	13%	7,561	4.7 million	Medium malnutrition; low IMR; high SWF	Argentina, Mexico, and Uruguay	3
**V5**	6%	3,123	1 million	Low malnutrition and IMR; low SWF	Peru, Chile and Brazil	2
**V2**	14%	6,690	14,741	Low malnutrition and IMR; low SWF	Chile, Peru and Brazil	1
**V6**	14%	8,479	2.2 million	Medium malnutrition; low IMR; highest SWF	Cuba, and Venezuela	1

### Comparative Coastal Risk Index (CCRI)–Equal weights scenario

Our results show that nearly 1.79 million people live in areas of high or very high CCRI (4.6 or 5, respectively) in LAC. This number represents almost 8% of the 23 million people population in the study area. Roughly 560,000 people live in areas of maximum CCRI value equal 5. These are areas where the maximum scores for hazards, exposure, and vulnerability (all equal 5) coexist. Areas with the second highest CCRI value (i.e. 4.6), include a coastal population of 1.2 million people.

Brazil, the largest country in the study region, also contains the largest coastal population, more than 8.6 million people. Mexico and Argentina have the second and third largest coastal populations (2.9, and 2.7 million people, respectively). However, the largest populations in areas of maximum CCRI (equal to 5) are in Ecuador (222,404 people), Mexico (130,810 people), and El Salvador (91,965 people).

The total coastal population in LAC in areas of maximum CCRI, (more than 566,000 people) are spread in 223 coastal segments, across 29 provinces in seven countries: Ecuador, Mexico, El Salvador, Honduras, Nicaragua, Guatemala, and Peru. Ecuador, Mexico, El Salvador and Honduras hold 87% of the total coastal population (494,330 people) and 80% of the number of coastal segments (179 segments) in areas of maximum CCRI. The 29 provinces in areas of maximum CCRI are located in countries in Northern, Central, and South America, facing the Pacific Ocean.

The roughly 560,000 people in areas of maximum CCRI values are distributed as follows: The majority (98.8%, or almost 560,000 people) belong to cluster H1, characterized by El Niño induced sea levels. Roughly 66% (372,000 people), belong to exposure cluster E6, characterized by large population and ecosystems. Roughly 32% belong to areas in cluster E8, characterized by large population, ecosystems and croplands; and 2.3% live in areas assigned to cluster E1, characterized by large population, GDP, and urban areas. Finally, 68.1% of the coastal population belong to cluster V9 (386,000 people), and almost 32% in cluster V1 (181.000 people). Both V1, and V9 clusters are characterized by high IMR, malnutrition, and low SWF. Cluster V1, however, has the highest IMR in the study (62 deaths per 1,000 births on average).

**Fig 5:**
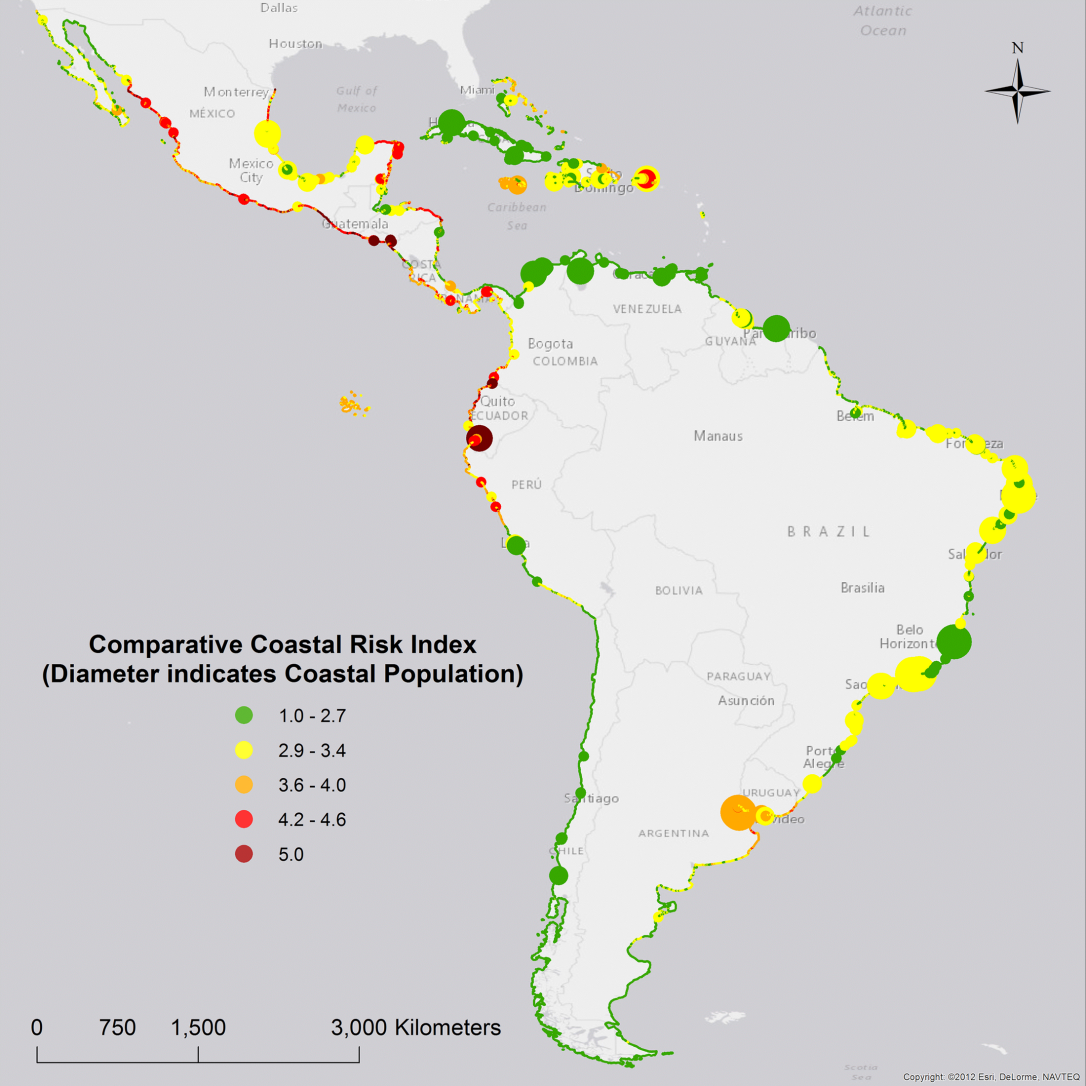
Comparative Coastal Risk Index (CCRI).

See Fig 5, and Table 7, for a geographic distribution of CCRI in LAC.

**Table 7:** Coastal population living in areas of maximum CCRI (value of 5).

Country	Locality	Coastal Population
**Ecuador**	El Oro	164,623
	Esmeraldas	38,170
	Manabi	17,442
	Guayas	2,169
	**Total Ecuador**	**222,404**
**Mexico**	Sinaloa	51,936
	Chiapas	44,709
	Nayarit	17,262
	Oaxaca	10,564
	Guerrero	6,339
	Tamaulipas	1,464
	**Total Mexico**	**132,274**
**El Salvador**	Usulutan	51,404
	La Paz	17,641
	La Union	11,092
	Ahuachapan	8,569
	Sonsonate	2,259
	**Total El Salvador**	**90,965**
**Honduras**	Choluteca	22,905
	Valle	20,683
	Gracias a Dios	4,858
	Colon	241
	**Total Honduras**	**48,687**
**Nicaragua**	Chinandega	35,988
	Carazo	714
	Zelaya	184
	**Total Nicaragua**	**36,886**
**Guatemala**	Santa Rosa	13,377
	Escuintla	10,172
	Jutiapa	3,935
	Retalhuleu	1,856
	**Total Guatemala**	**29,340**
**Peru**	Piura	3,794
	Ancash	2,059
	La Libertad	319
	**Total Peru**	**6,172**
	**Grand Total**	**566,728**

### Hotspots (areas of maximum CCRI = 5)

**Fig 6:**
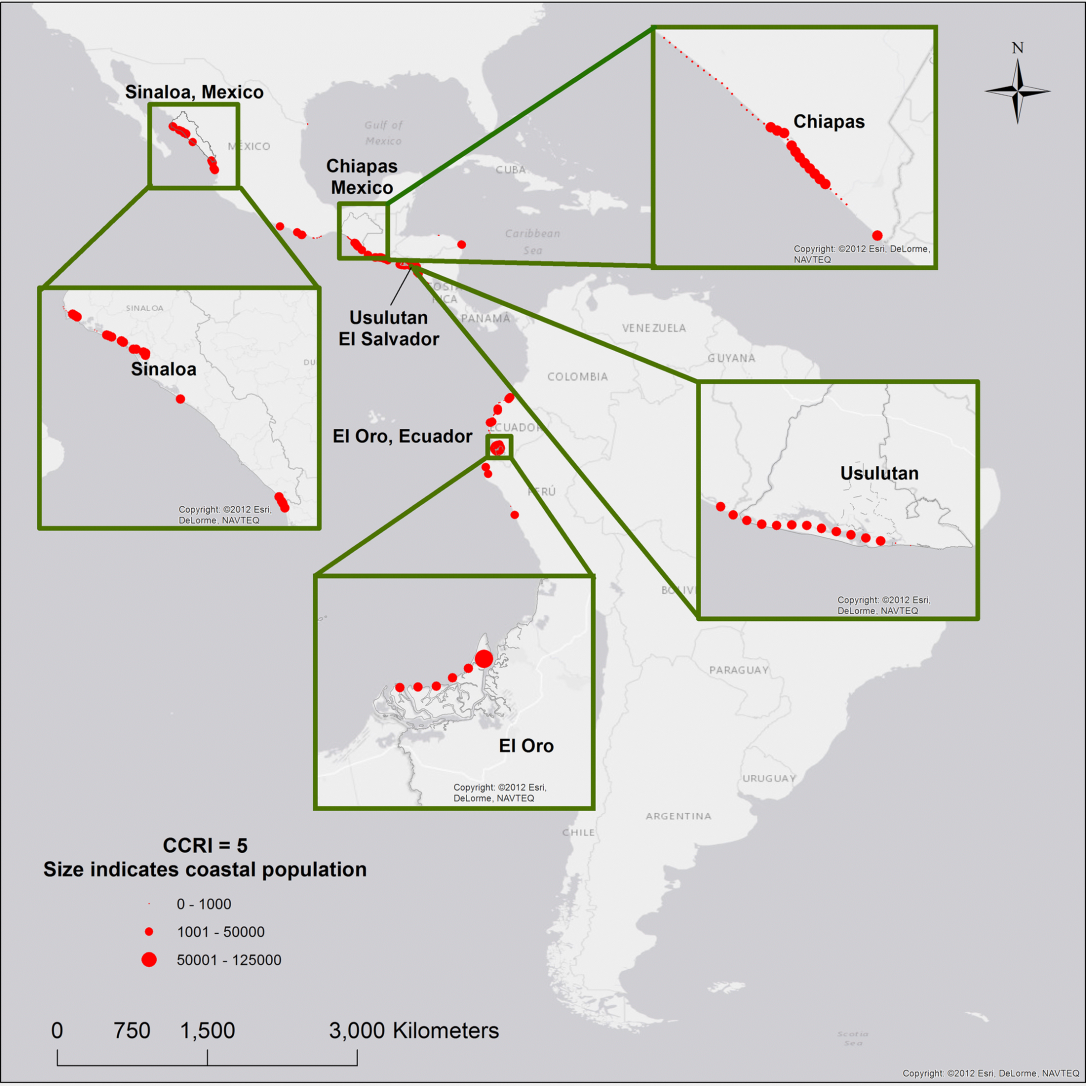
Hotspots of coastal risk.

Our results show that 55% of the coastal population (more than 310,000 people) living in areas of maximum CCRI, are concentrated in four provinces: El Oro, Ecuador; Sinaloa and Chiapas, Mexico; and Usulutan, El Salvador (Fig 6).

El Oro, Ecuador: flood events are a yearly occurrence in El Oro, and are usually intensified during strong El Niño events [66]. The 1997/1998 El Niño resulted in major economic and social impacts to the province, severely damaging more than 2,000 residences [66], and resulting in almost 300 cases of Malaria and 200 cases of Dengue fever [67]. During the 1982/1983 El Niño, infant mortality rates in flood affected areas in El Oro increased 16% (from 52 to 65 per thousand live births) [67].

Sinaloa, Mexico: in 2013, Hurricane Manuel made landfall in Sinaloa, with wind speeds reaching 150km/h [68]. The Hurricane caused more than $4 billion in damages, and 123 deaths in Mexico [69]. In the fishing village of Yameto, Sinaloa, a coastal segment classified with high CCRI (score equal to 4.6), 60 families were evacuated during the hurricane [70]. In 2014, almost 40% of the population of Sinaloa were living in poverty [71].

Chiapas, Mexico: the 1997/1998 El Niño resulted in more than 200 deaths and caused severe damages to more than 15 coastal counties in Chiapas, isolating nearly 500,000 people for several days [72]. Infant mortality rates in Chiapas, were three times higher than the Mexican average in 2010 [73]. More recently, in 2014, 76.2% of the population of Chiapas lived below the poverty line, and 31.8% of the population lived in extreme poverty [71].

Usulutan, El Salvador: roughly 66% of all disasters in El Salvador, between 1990 and 2014, were related to floods and tropical storms [74]. In 2009, in the Usulutan province, where 75% of all coastal segments were classified as very high CCRI (scores equal to 5), 37.4% of the population lived below the poverty line, and 11.6% lived in extreme poverty [75]. More than 3,200 houses in the province were destroyed during the 1987/1988 El Niño [76].

Ranked by coastal population, the analysis below focuses on the four areas above, where the three dimensions of coastal risk (hazards, exposure, and vulnerability) have maximum scores (equal to 5).

#### 1. El Oro, Ecuador

Ecuador contains the largest coastal population in the study area with CCRI index value equal 5 (222,404 people). El Oro, the southernmost coastal Province of Ecuador, contains almost 30% of that population (164,623 people), concentrated in six coastal segments. The most relevant drivers of coastal risk in El Oro are:

- **Hazards**: all six coastal segments in El Oro belong to cluster H1, with El Niño as the main driver of coastal hazards;
- **Exposure**: five coastal segments belong to cluster E8, characterized by large population, ecosystems and croplands; one coastal segment belongs to cluster E6, characterized by large population and ecosystems.
- **Vulnerability**: five coastal segments belong to cluster V9, and one coastal segment belongs to cluster V1. Both V1 and V9 clusters are characterized by high IMR (35 deaths per thousand births), high malnutrition rates (14.8%), and low SWF. However, cluster V1 has much lower SWF values than V9.

#### 2. Sinaloa, Mexico

Mexico contains the second largest coastal population in the study area with maximum CCRI value of 5 (132,274 people). The state of Sinaloa, on the Gulf of California, contains roughly 39% of that total (51,936 people) concentrated in 24 coastal segments. The most relevant drivers of coastal risk in Sinaloa are:

- **Hazards**: All 24 coastal segments in Sinaloa belong to cluster H1, with El Niño as the main driver of coastal hazards.
- **Exposure**: 21 coastal segments belong to cluster E6, characterized by large population and ecosystems; the 3 remaining coastal segments belong to cluster E1, characterized by large population, GDP, and urban areas.
- **Vulnerability**: All 24 coastal segments in Sinaloa belong to cluster V9, characterized by high IMR (24.3 deaths per thousand births), high malnutrition rate (7.5%), and low SWF values.

#### 3. Usulutan, El Salvador

El Salvador contains the third largest coastal population in the study area with maximum CCRI value of 5 (90,965 people). The province of Usulutan, in the southeast region of El Salvador (facing the Pacific Ocean), contains 57% of that population (51,404 people), concentrated in 12 coastal segments. The most relevant drivers of coastal risk in Usulutan are:

- **Hazards**: All 12 coastal segments in Usulutan belong to cluster H1, characterized by El Niño as the main driver of coastal hazards.
- **Exposure**: All the 12 coastal segments in Usulutan belong to cluster E6, characterized by large population and ecosystems.
- **Vulnerability**: All 12 coastal segments in Usulutan belong to cluster V9, characterized by high infant mortality rate (31.2 per thousand births), high malnutrition rate (11.3%), and low SWF values.

#### 4. Chiapas, Mexico

The state of Chiapas, on the shoreline of the Gulf of California, contains 35 coastal segments with 44,709 people living in areas of maximum CCRI. This represents roughly 33.8% of the total population in maximum CCRI areas in Mexico. The most relevant drivers of coastal risk in Chiapas are:

- **Hazards**: All 35 coastal segments in Chiapas belong to cluster H1, characterized by El Niño as the main driver of coastal hazards;
- **Exposure**: All 35 coastal segments belong to cluster E6, characterized by large population and ecosystems;
- **Vulnerability**: All 35 coastal segments in Chiapas belong to cluster V9, characterized by high infant mortality rate (31.9 deaths per thousand births), high malnutrition rate (7.5%), and low SWF values.

### Discussion

As coastal populations increase around the globe, the combination of coastal hazards, geographic exposure and socioeconomic vulnerability can greatly intensify coastal risks. In a vicious cycle, the occurrence of disasters leads to a reduction of income and consumption levels, aggravating poverty, and limiting the population’s ability to minimize and cope with future impacts.

Our results show that in LAC, more than 500,000 people live in areas of maximum CCRI, with more than 310,000 people concentrated in four hotspot locations: El Oro, Ecuador; Sinaloa and Chiapas, Mexico; and Usulutan, El Salvador (Fig 6). These are communities where scarce critical resources are consistently placed in hazards prone areas further exacerbating risks and impacts from coastal hazards.

Notably, some areas considered hotspots of coastal exposure in previous studies, including a number of Caribbean islands, and Rio de La Plata [16,29], do not peak within CCRI. Several areas of the Caribbean received maximum hazards scores (e.g. The Bahamas) and maximum vulnerability scores (i.e. Haiti). However, except for very few coastal areas (e.g. Havana, Cuba), exposure scores in The Caribbean ranged from 2 to 3, driving lower CCRI values. Similarly, coastal segments in the Rio de la Plata region received maximum coastal hazards scores, but did not receive highest vulnerability and hazards scores, resulting in CCRI values from 2.9 to 4.3. Nevertheless, coastal risk affects areas beyond those where CCRI equals 5. Particularly, areas where CCRI values are equal or greater than 4 include an additional 1.6 million people, and should also be prioritized. Such areas did not peak in the CCRI index due to the variables selected and the equal weight scheme utilized in the calculations. If variables are substituted, or if individual scores are weighted differently, the results are likely to change.

While the impacts from climate change are not part of the scope of the present study, it is important to acknowledge that they pose additional threats to coastal areas [20]. Climate change impacts, including more frequent high-intensity storms, higher sea-levels, and more severe floods will pose additional challenges to coastal communities [4,26,77]. Global sea-level rise projections for the year 2100 range from 81cm to 179cm, which will lead to more frequent and widespread coastal flooding [78–81]. Nuisance floods–minor, recurrent flooding that takes place at high tide–already cause frequent road closures, overwhelm storm water drainage, having a non-linear impact on critical infrastructure [82,83].

Despite recent efforts to assess coastal risks in a multidisciplinary way, further research is still needed. The methods proposed here can be enhanced by the introduction of temporal variability via the addition of future projections (e.g. population growth, land use, and hazards projections). Additionally, a panel of experts could be convened to review the input variables and weighting of CCRI. Finally, higher resolution, small scale studies, focused on coastal risk reduction are needed.

The techniques employed here provide a robust toolset to identify patterns through multivariate and complex datasets. The benefits of the proposed approach are multiple and include: reduction of multiple independent variables into a single coastal risk index; the identification of major drivers of coastal risk and related hotspots; the ability to identify coastal areas seemly unrelated, but facing very similar challenges and may benefit from future collaborations to reduce coastal risk.

Moreover, the current study can inform coastal policies. Coastal risks reduction and adaptation efforts must not only focus on developing coastal defenses, but also on improving practices and policies related to urban development and zoning, agriculture, and conservation, as well as on ameliorating socioeconomic conditions. Policies including restoration and preservation of natural habitat, and agricultural practices, should also be considered. As an example, the conservation and restoration of coastal habitats, which may act as coastal defenses to natural hazards, can also improve fisheries, positively impacting the livelihoods of local fishing communities reducing their vulnerability.

The implementation of a sisters-city like approach (where cities, or provinces, form partnerships to promote cultural and commercial ties) should also be considered. Coastal communities of similar coastal risk profiles, can greatly benefit by an exchange of experiences and lessons learned from past disasters, coastal adaptation projects, and coping mechanisms.

## Supporting information

S1 Appendix Various outputs from Self-Organizing Maps and K-Means algorithms. (PDF)

S2 Appendix Complete list of data sources. (PDF)
