# Supplementary material for: Comparative Coastal Risk Index (CCRI): A multidisciplinary risk index for Latin America and the Caribbean
Source: PLoS One. 2017 Nov 2;12(11):e0187011. doi: 10.1371/journal.pone.0187011 (PMC5667813; doi:10.1371/journal.pone.0187011)
Supplement: S1 Appendix — (PDF) [file pone.0187011.s001.pdf]

Hazards – Self Organizing Maps (10X10 matrix) for each Hazard variable with probability matrix

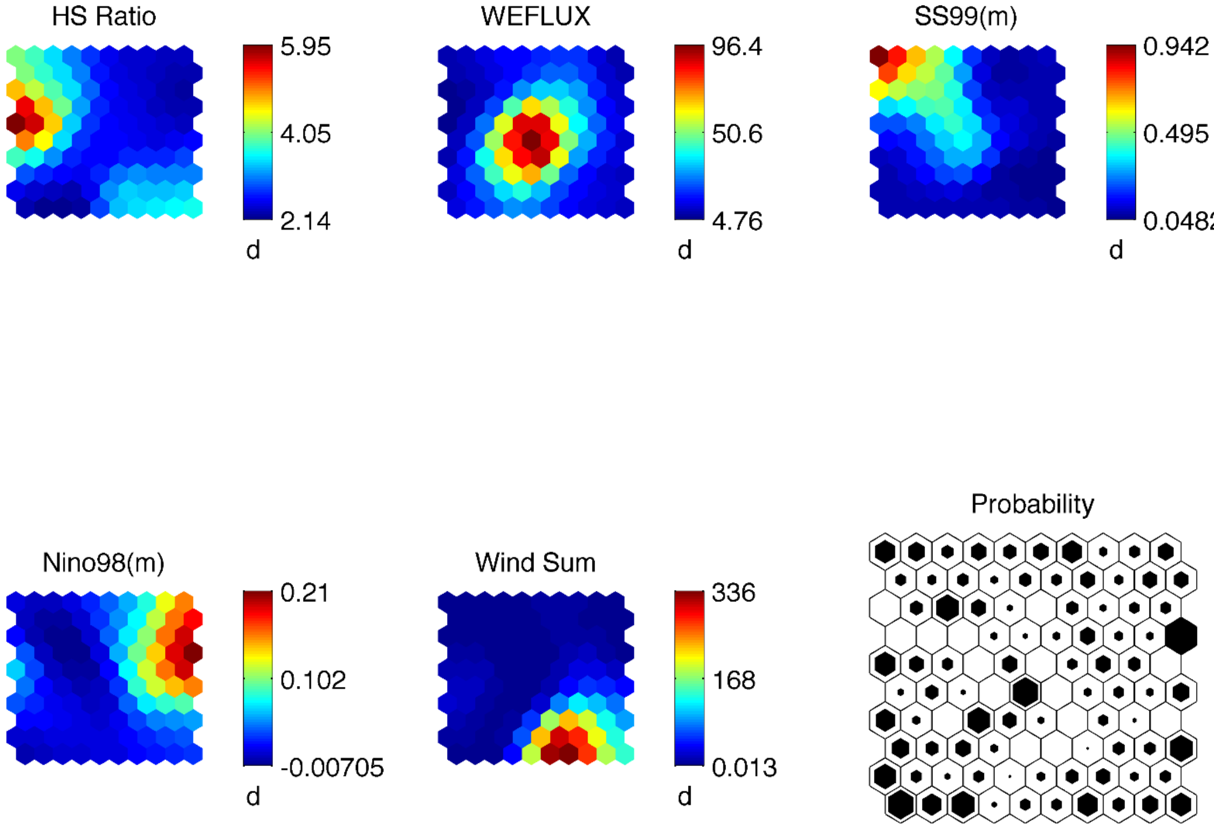

SOM 04-Nov-2016

9 K-Means Clusters (Indicated by distinct colors)

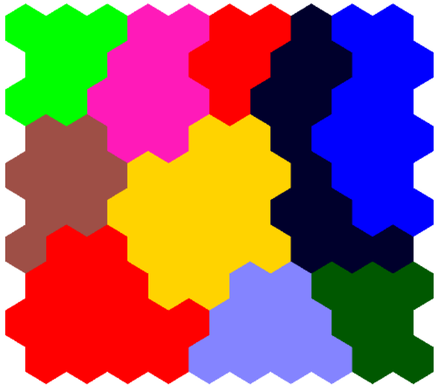

Hazards – Self Organizing Maps (10X10 matrix) for each Hazard variable with probability matrix

9 K-Means Clusters

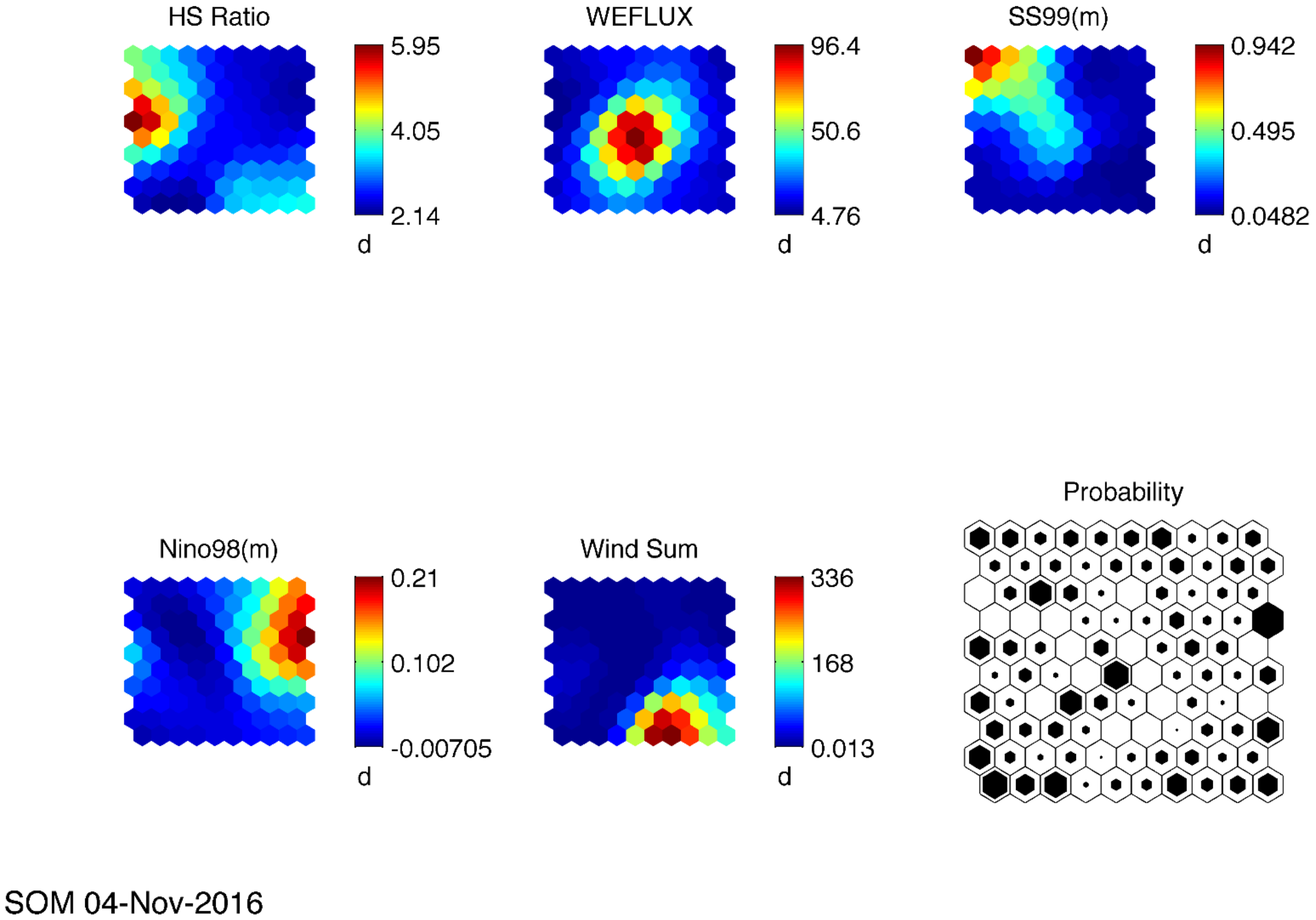

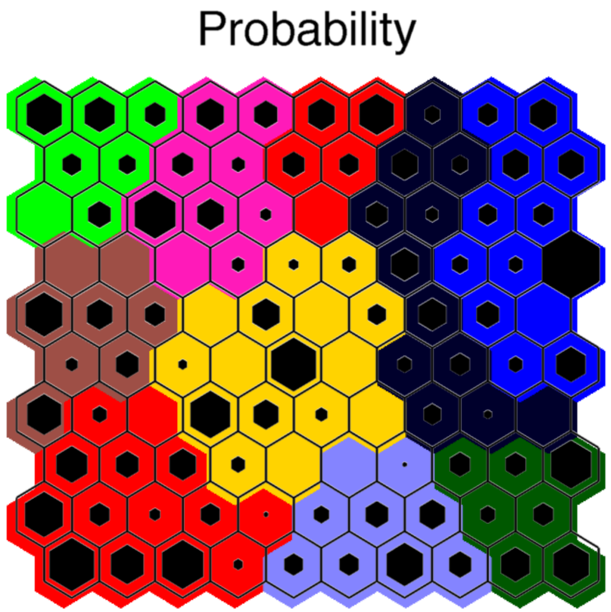

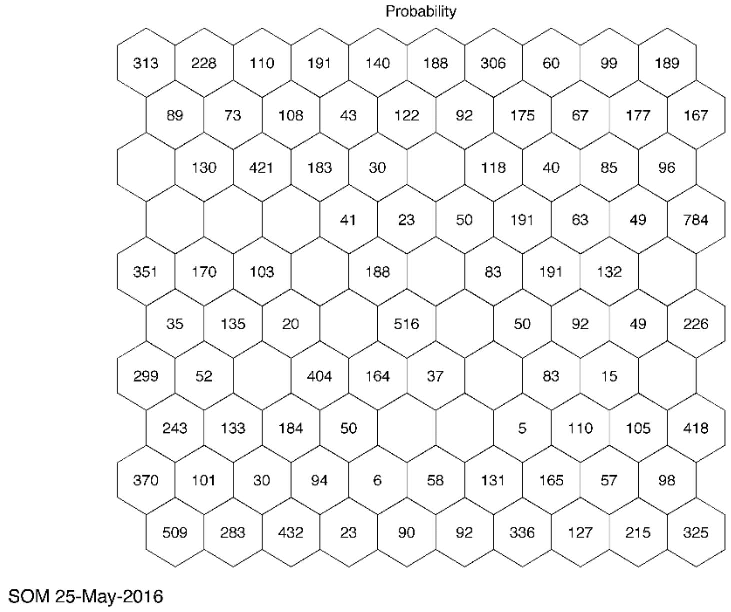

9 K-Means Clusters - Hazards

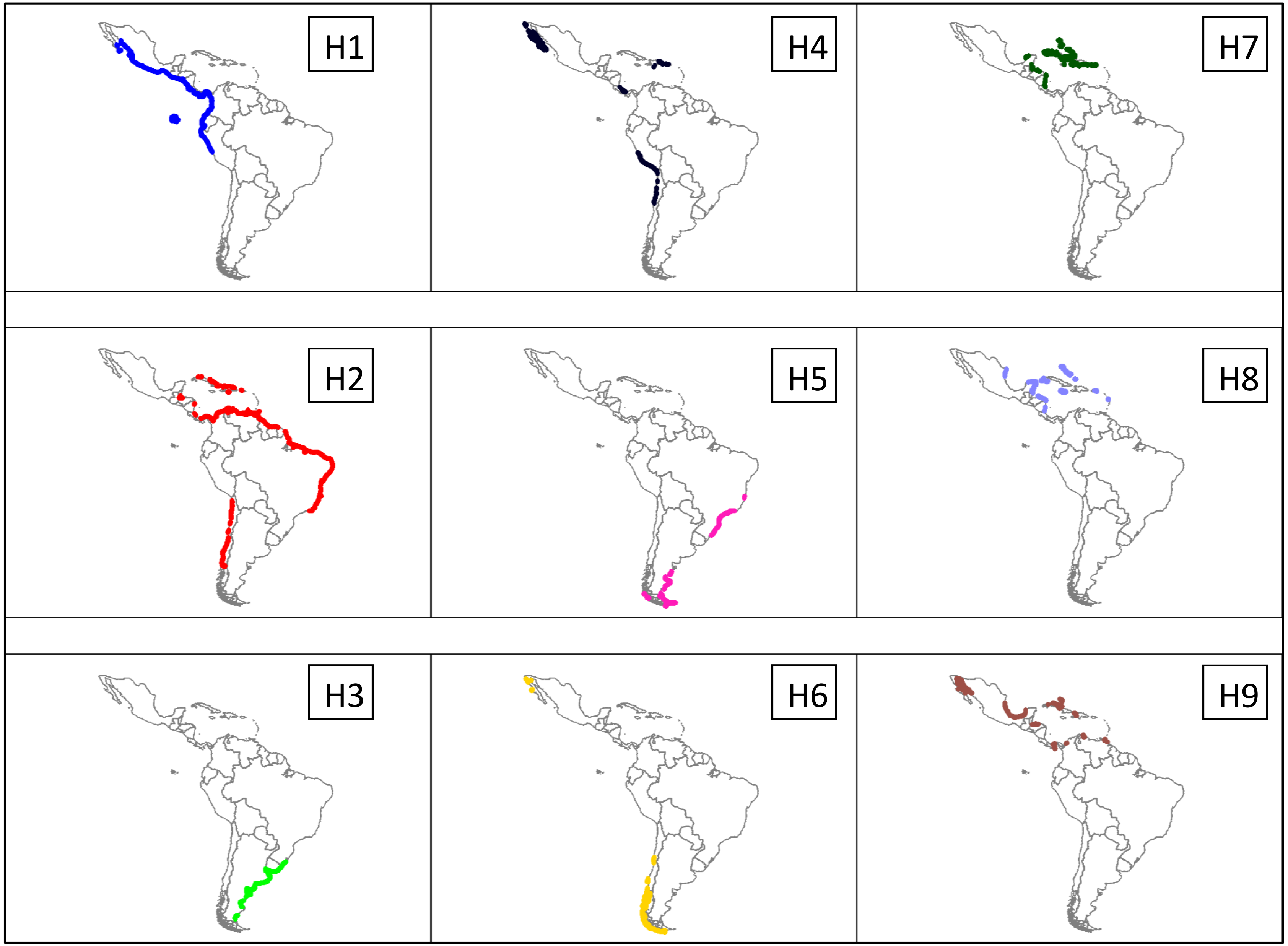

Cluster's Statistics (normalized from 0 to 1)

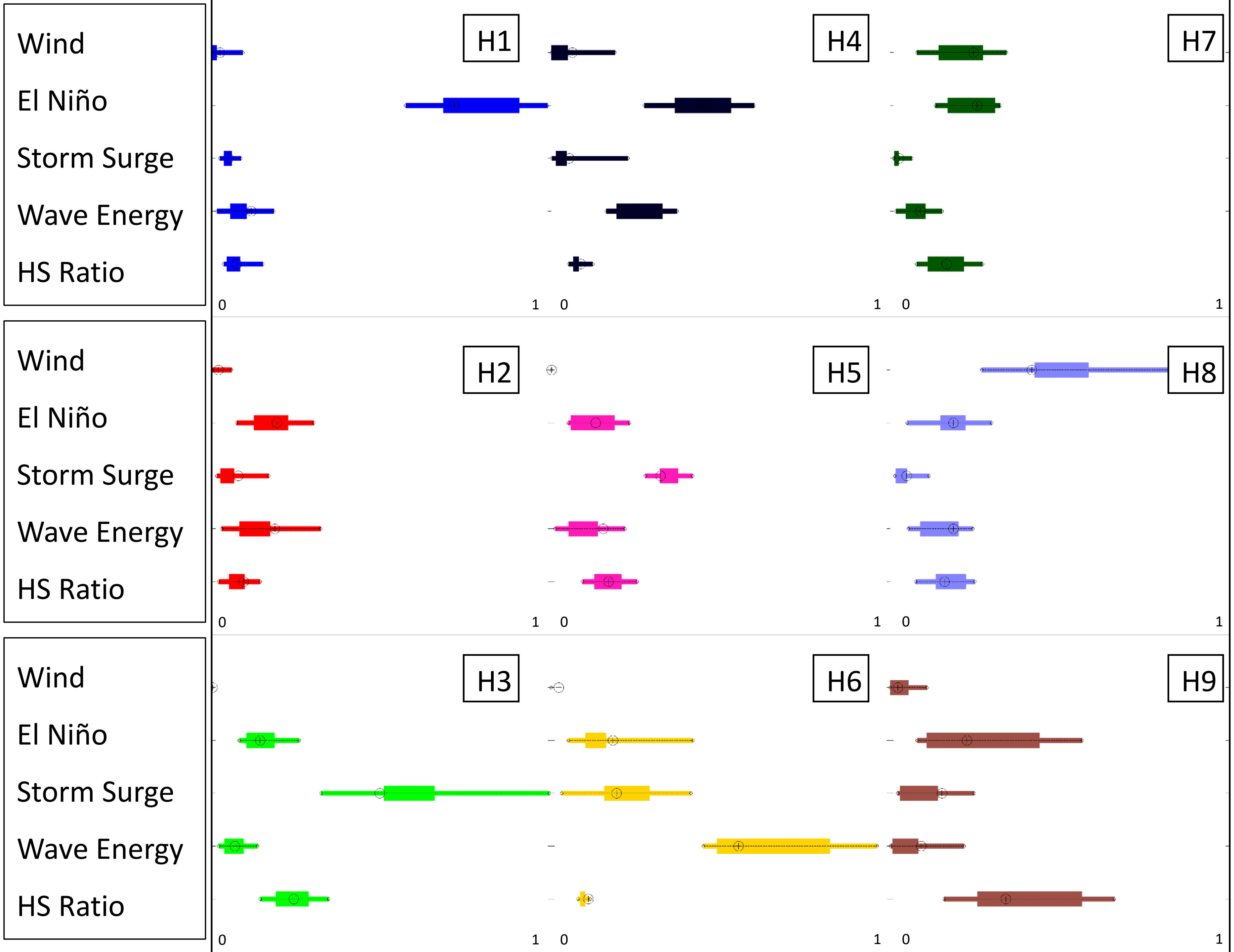

Sample: K-Means Cluster Hazards Statistics

K-Means	N Rows	Mean(Wave Energy)	Histogram(Wave Energy)	Mean(SS99)	Histogram(SS99)	Mean(El Nino)	Histogram(El Nino)	Mean(Wind Sum)	Histogram(Wind Sum)	Mean(HS Ratio)	Histogram(HS Ratio)
1	2116	0.0858696969		0.0478584344		0.7825268029		0.0172924252		0.071056078	
2	3168	0.1346826448		0.0544004294		0.1767813514		0.0097777377		0.0744972804	
3	943	0.0682698695		0.5905037952		0.1401242673		0		0.2337813691	
4	1082	0.2678500391		0.0499076808		0.445247803		0.0343851505		0.076743854	
5	1157	0.1028690753		0.3439554035		0.1208060182		0		0.1698981274	
6	1535	0.6644418599		0.2211877062		0.1475840497		0.000024144		0.094377563	
7	1328	0.0766396739		0.0238296092		0.2461370753		0.2062254648		0.1718667528	
8	1004	0.1390754709		0.0394996769		0.178700361		0.5176629101		0.1767546375	
9	1093	0.0606986355		0.1004138202		0.2800525827		0.033175051		0.3953525411	

Exposure - Self Organizing Maps (10X10 matrix) for each Exposure variable with probability matrix

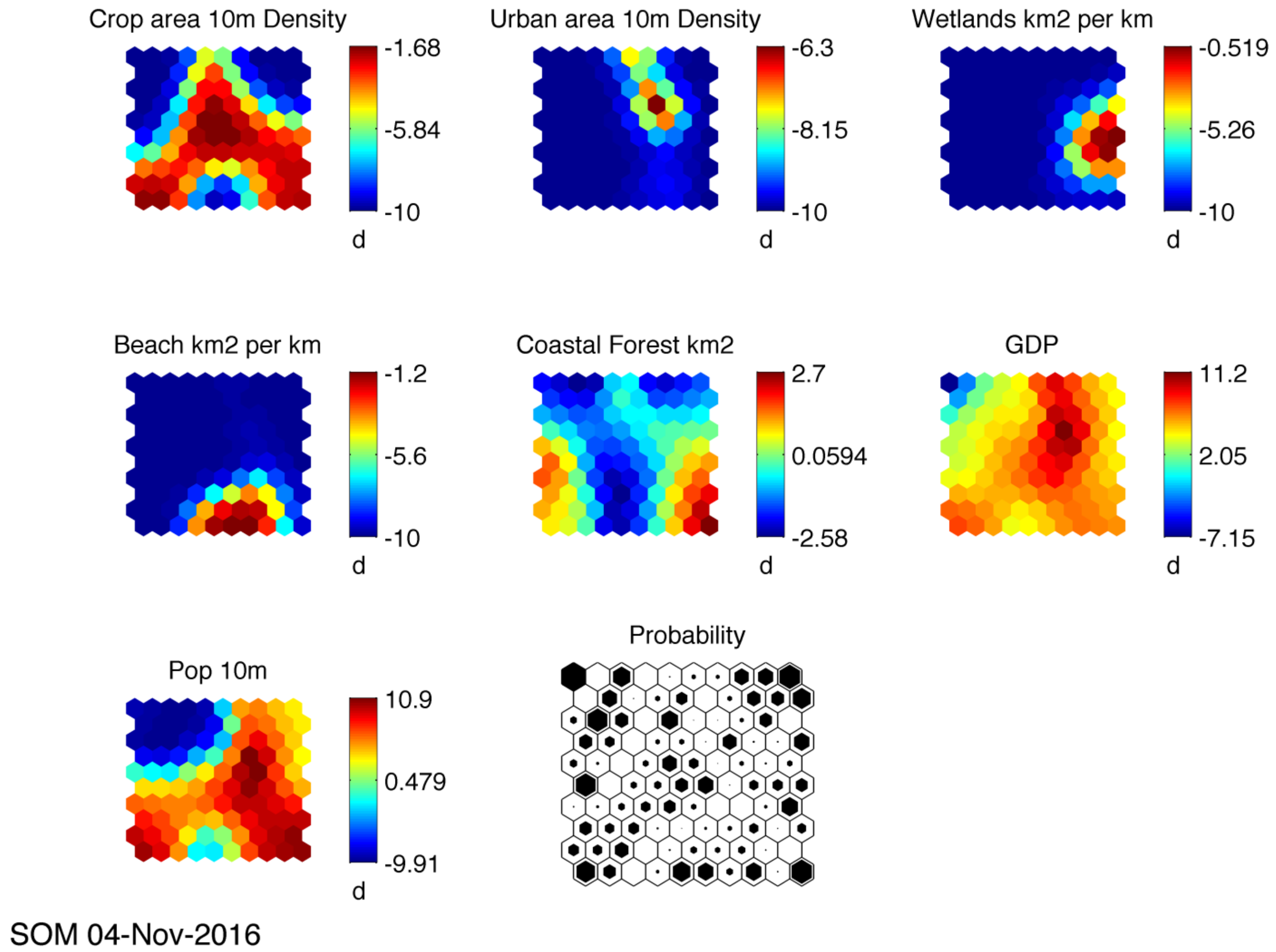

9 K-Means Clusters (Indicated by distinct colors)

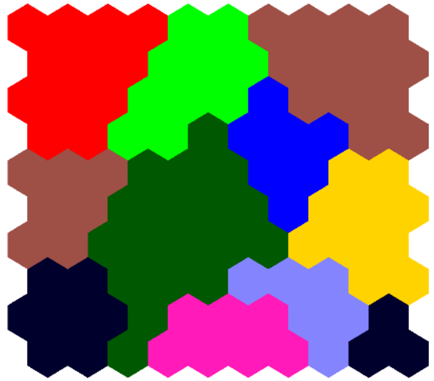

Exposure - Self Organizing Maps (10X10 matrix) for each Exposure variable with probability matrix

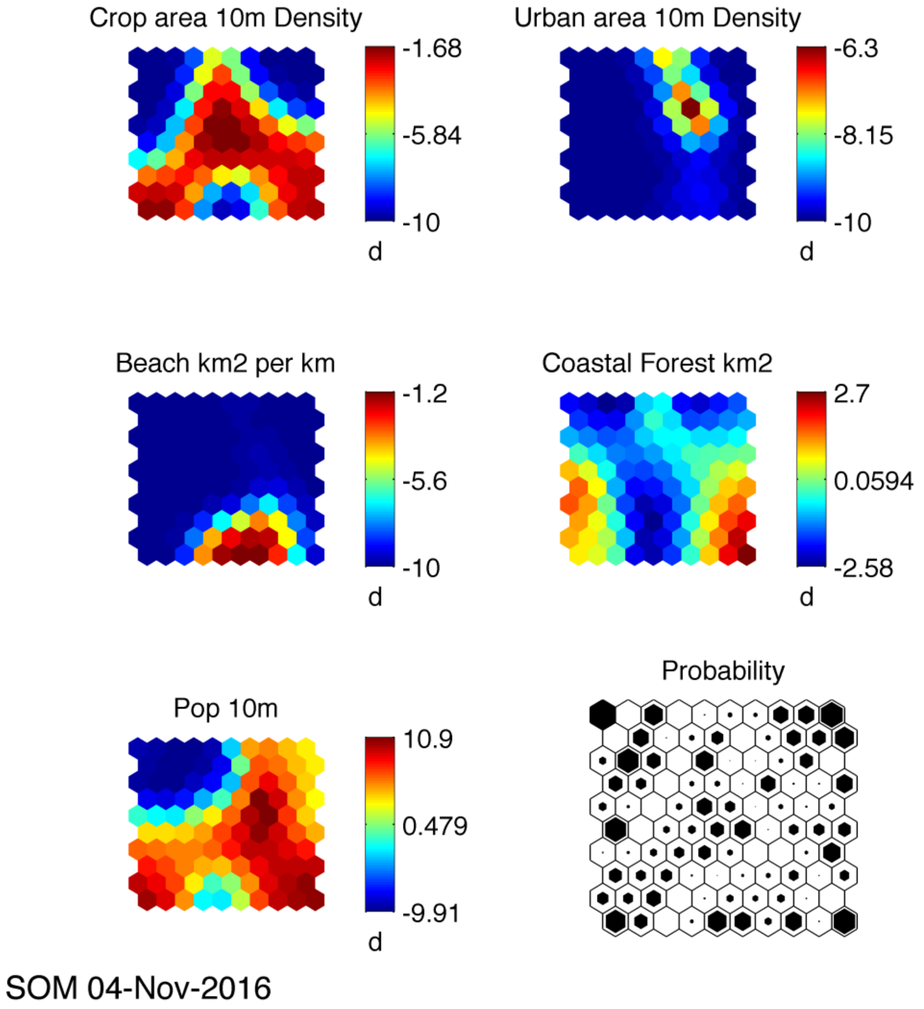

9 K-Means Clusters (Indicated by distinct colors)

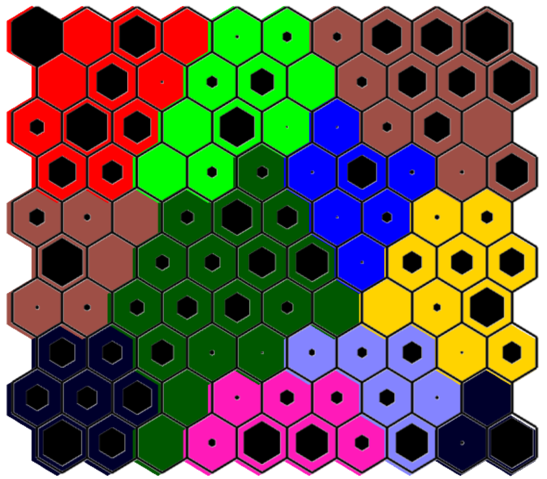

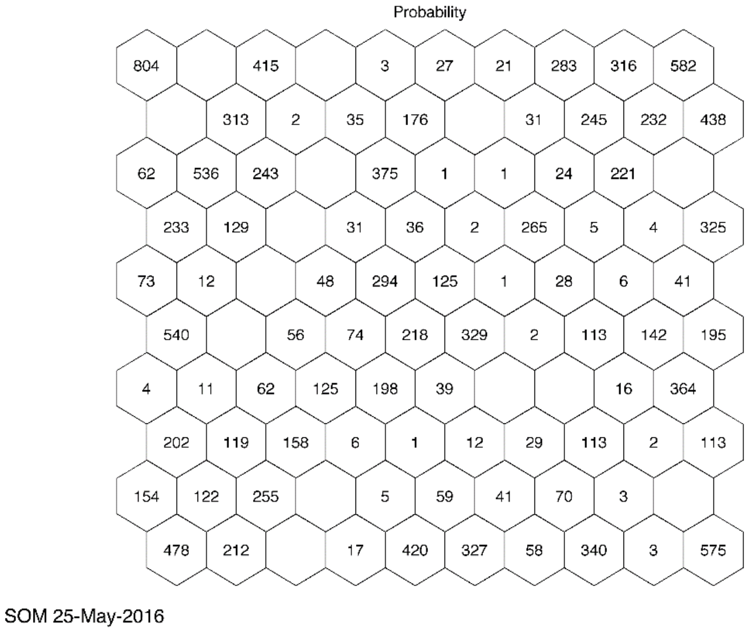

9 K-Means Clusters - Exposure

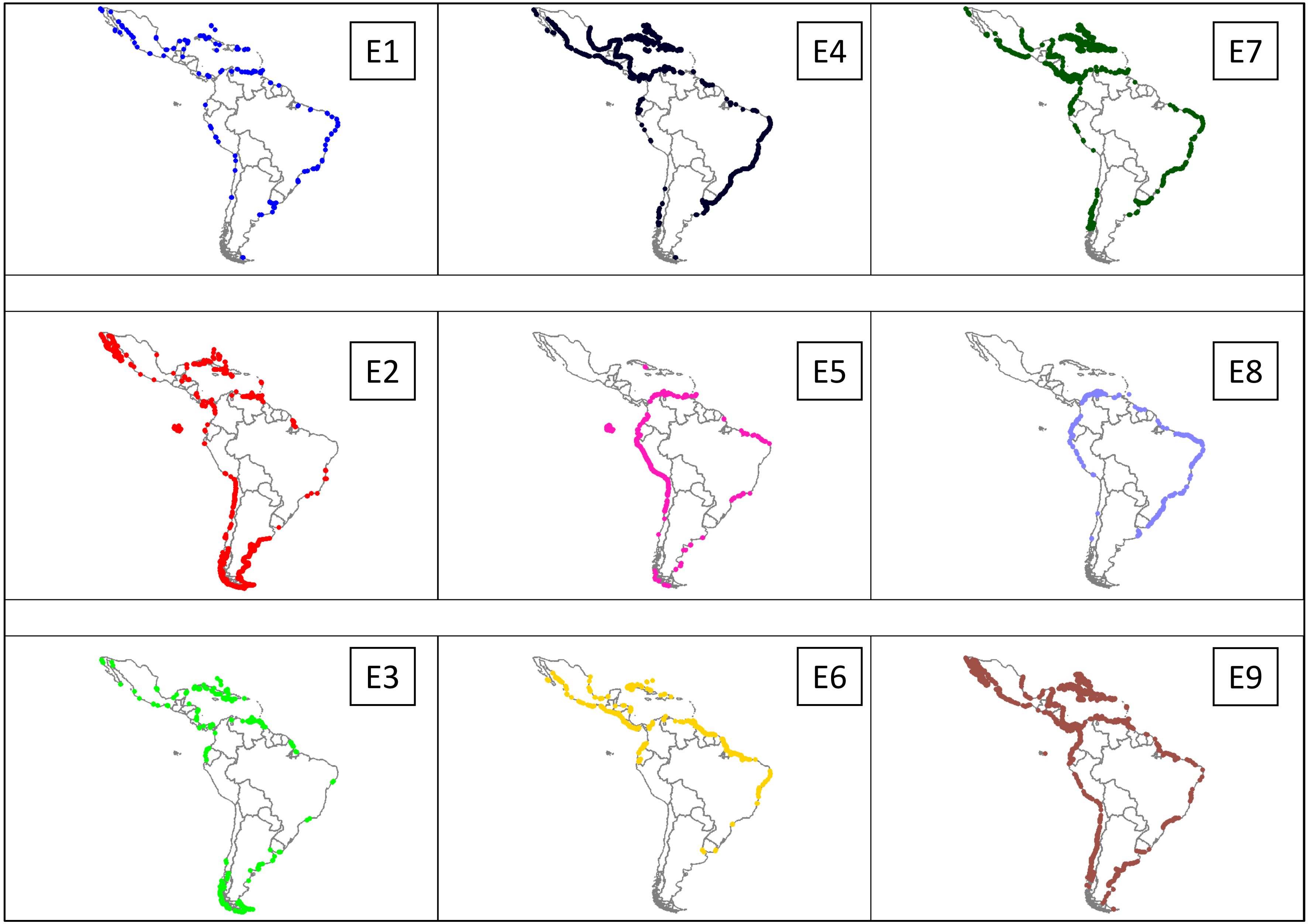

Cluster's Statistics (normalized from 0 to 1)

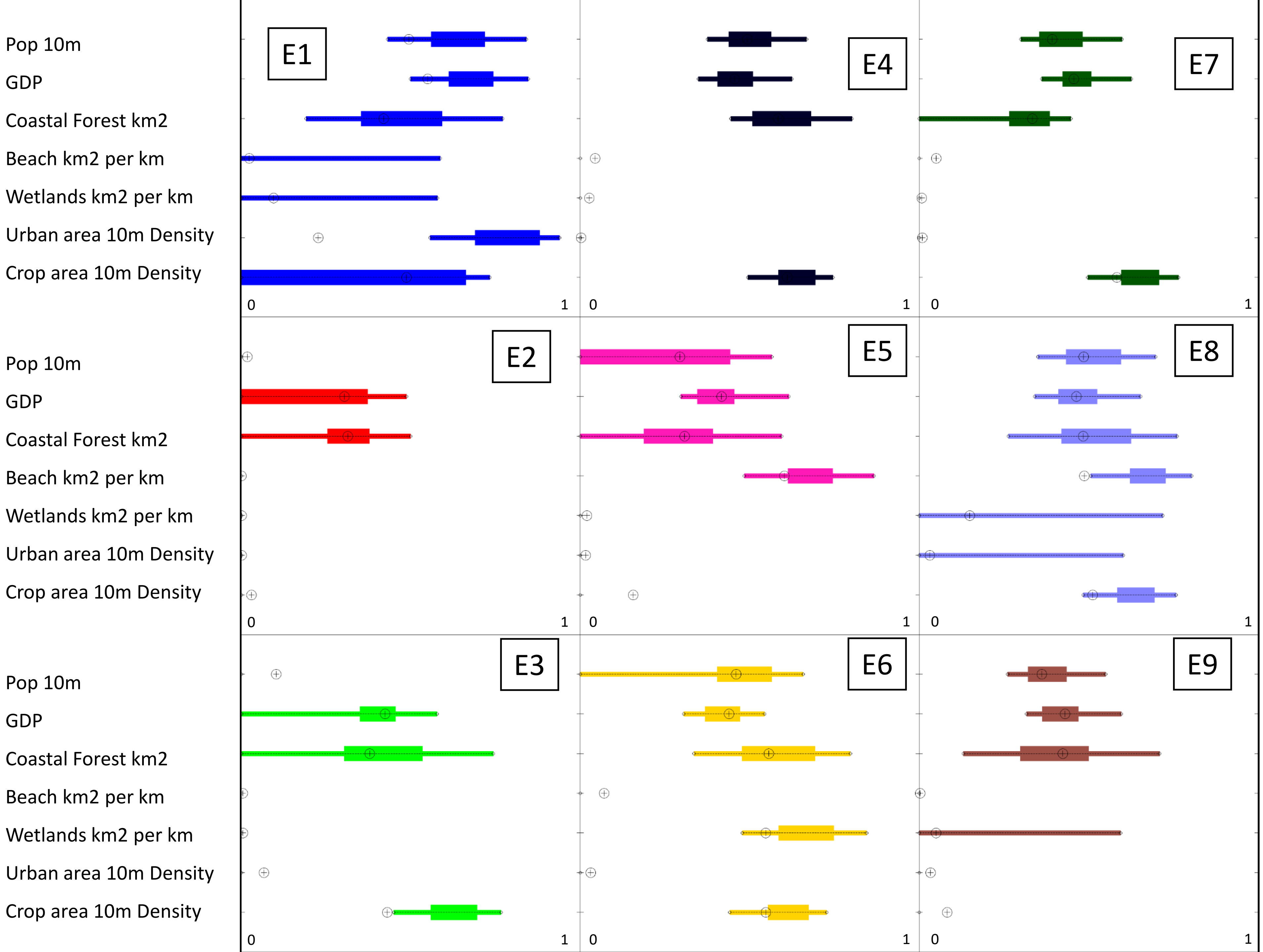

Vulnerability - Self Organizing Maps (10X10 matrix) for each vulnerability variable with probability matrix

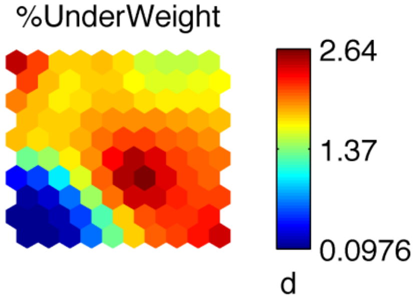

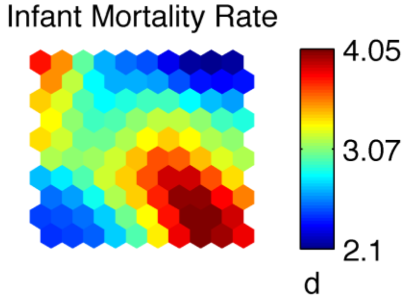

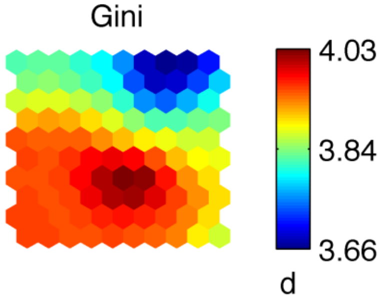

9 K-Means Clusters (Indicated by distinct colors)

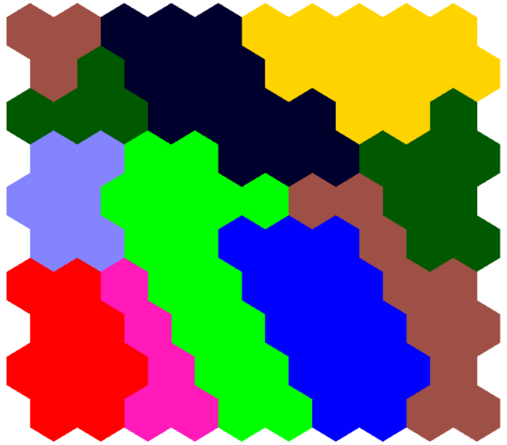

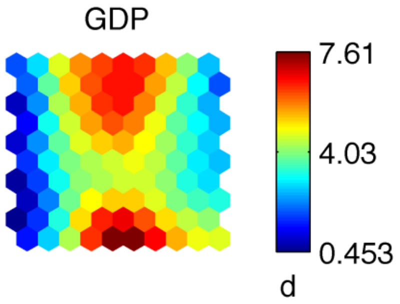

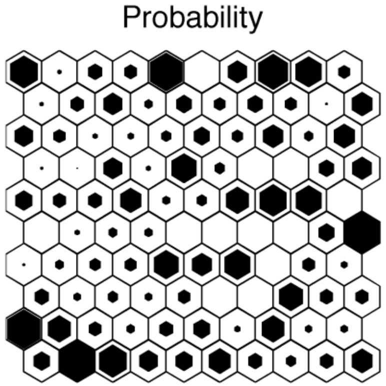

SOM 12-Dec-2016

Vulnerability - Self Organizing Maps (10X10 matrix) for each vulnerability variable with probability matrix

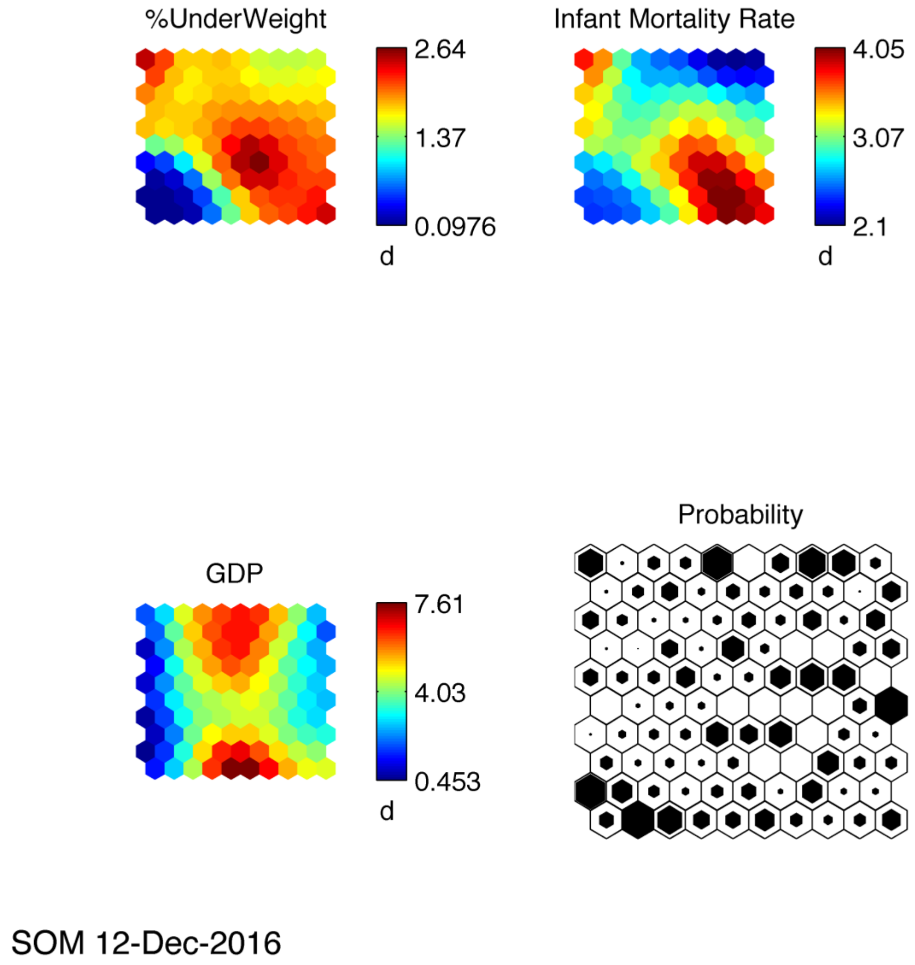

9 K-Means Clusters (Indicated by distinct colors)

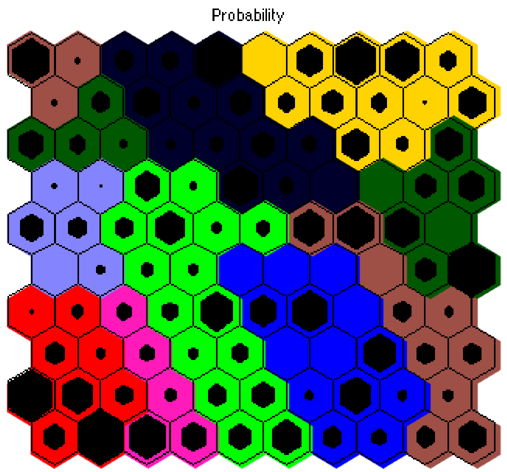

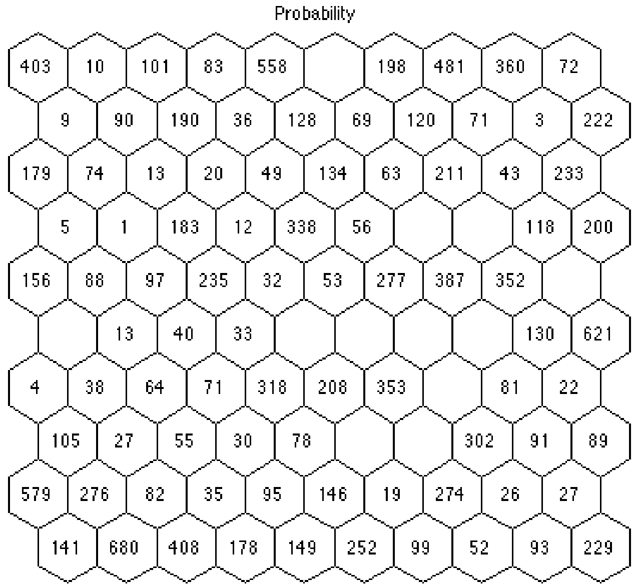

9 K-Means Clusters - Vulnerability

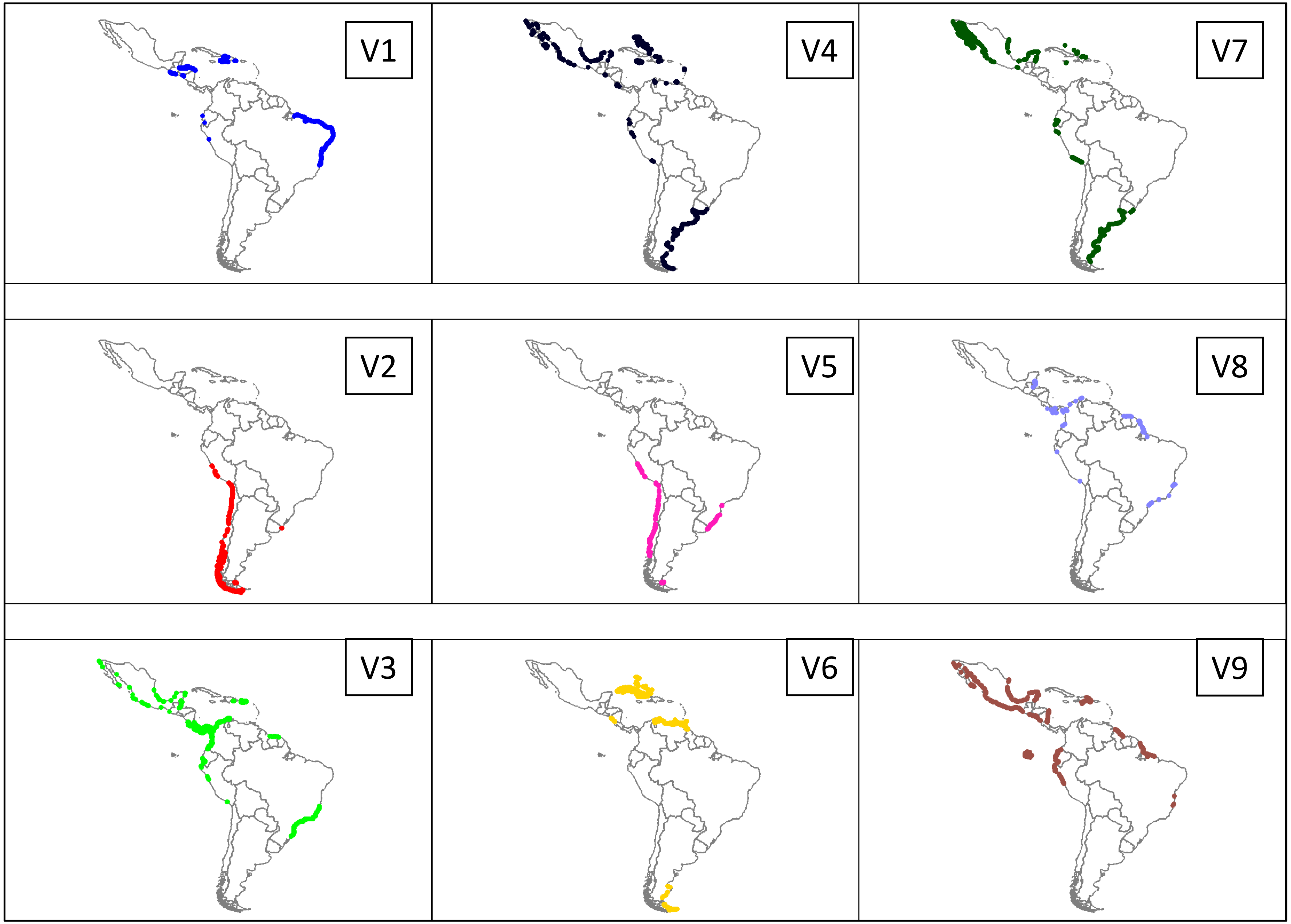

Cluster's Statistics (normalized from 0 to 1)

GDP

Gini

Infant Mortality Rate

% Underweight

GDP

Gini

Infant Mortality Rate

% Underweight

GDP

Gini

Infant Mortality Rate

% Underweight

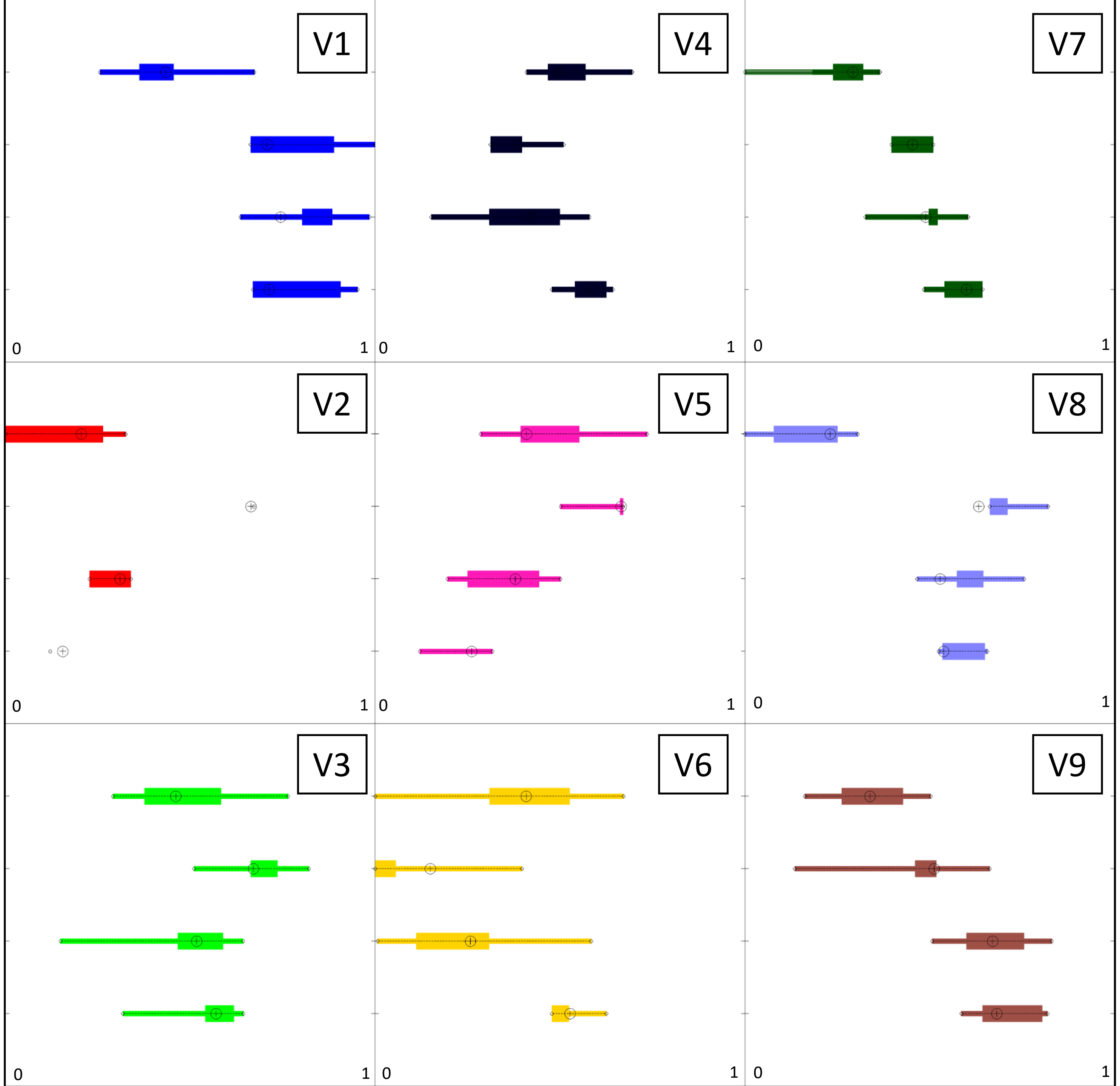

Sample K-Means Cluster Vulnerability

K-Means	N Rows	Mean(%UnderWeight)	Mean(Mortality)	Mean(GINI)	Mean(GDP)	Histogram(%Under Weight)	Histogram(Mortality)	Histogram(GINI)	Histogram(GDP)
1	1333	0.775965115789621	0.83551802438239	0.7741100689272	0.420505408063459	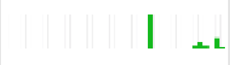	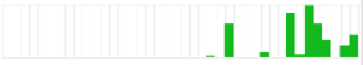	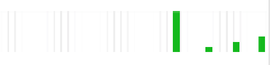	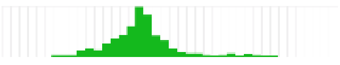
2	1932	0.12238828603617	0.27614799282587	0.66556779535026	0.172994705666514	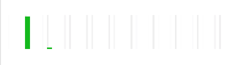	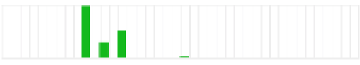	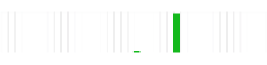	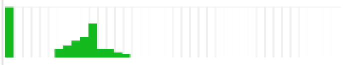
3	1824	0.56572645268627	0.49595990197717	0.68581040930024	0.484473707926215	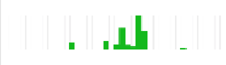	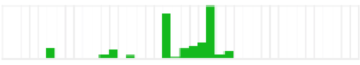	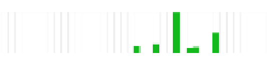	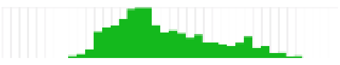
4	1756	0.578658767342739	0.39450833110784	0.38106520599262	0.528088485792487	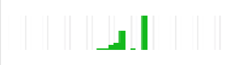	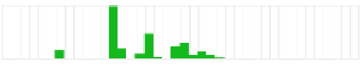	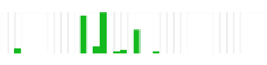	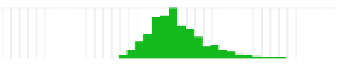
5	740	0.162405611974409	0.33246643892461	0.64347008988118	0.474565679066443	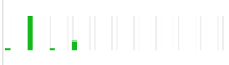	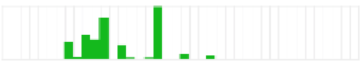	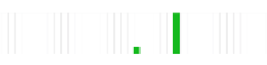	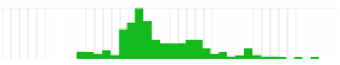
6	1850	0.510923118300054	0.21461937559722	0.07274946957275	0.397275112570467	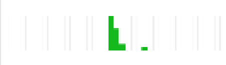	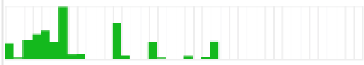	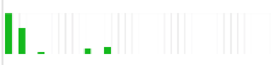	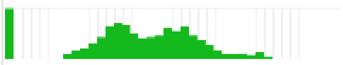
7	2010	0.601273615698558	0.48418782220538	0.46969871678826	0.263047314728395	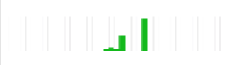	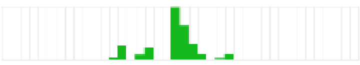	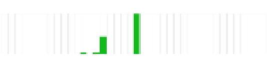	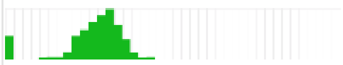
8	263	0.594335750273435	0.60371648858827	0.69340777505555	0.167295103742832	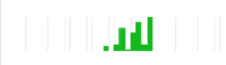	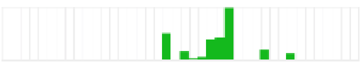	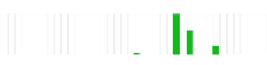	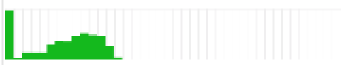
9	1718	0.704549675203073	0.67189378423161	0.47920903525086	0.352385563292248	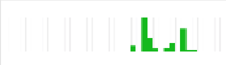	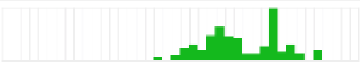	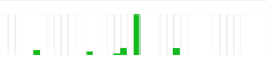	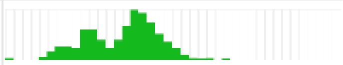
