# Supplementary material for: Comparative Coastal Risk Index (CCRI): A multidisciplinary risk index for Latin America and the Caribbean
Source: PLoS One. 2017 Nov 2;12(11):e0187011. doi: 10.1371/journal.pone.0187011 (PMC5667813; doi:10.1371/journal.pone.0187011)
Supplement: S2 Appendix — (PDF) [file pone.0187011.s002.pdf]

Supplemental Appendix 2 – Additional Data Sources

Most the socioeconomic data used in the study were downloaded from the "Socioeconomic Data and Applications Center" (sedac). However, data for a few select countries were not available and were complemented by other sources as described below:

1. Child Malnutrition (ratio between the number of children under five (the rate denominator), and the number of underweight children under five (the rate numerator).

Main Source (sedac):

http://sedac.ciesin.columbia.edu/data/set/povmap-global-subnational-prevalence-child-malnutrition

Additional data downloaded from other sources:

Haiti / Trinidad Tobago/ Dominica

http://www.who.int/gho/publications/world_health_statistics/EN_WHS10_Full.pdf

Suriname:

https://www.cia.gov/library/publications/the-world-factbook/fields/2224.html

Bahamas

https://books.google.com/books?id=RCZ1AwAAQBAJ&pg=PA61&lpg=PA61&dq=bahamas+percentage+of+underweight&source=bl&ots=NsVcSXiA-6&sig=4Re04-3Wgcc_uRfo4_Sh6FrqlRk&hl=en&sa=X&ei=kGvkVPH3GMPZoATI3YIY&ved=0CEwQ6AEwBw#v=onepage&q=bahamas%20percentage%20of%20underweight&f=false

Puerto Rico

http://www.ishib.org/ED/journal/21-2/ethn-21-02-163.pdf

2. Infant Mortality Rates (number of children who die before their first birthday for every 1,000 live births)

Main Source (sedac):

http://sedac.ciesin.columbia.edu/data/set/povmap-global-subnational-infant-mortality-rates

Additional data downloaded from other sources:

Aruba:

https://www.cia.gov/library/publications/the-world-factbook/fields/2091.html

Galapagos Islands:

http://islands.unep.ch/CKC.htm

French Guiana:

http://esa.un.org/wpp/Demographic-Profiles/pdfs/254.pdf

Venezuela (La Tortuga Island)

Assumed same Value as Venezuela (28 deaths / thousand live births)

Curacao:

https://books.google.com/books?id=xN7nNRr4lGIC&pg=PA403&lpg=PA403&dq=curacao+infant+mortality+rate&source=bl&ots=7Try0ba_di&sig=510l2PQUxhTlhzI5w6YI2HKmeXg&hl=en&sa=X&ei=gF3kVJW5Bs_voAT8n4C4CA&ved=0CD4Q6AEwAw#v=onepage&q=curacao%20infant%20mortality%20rate&f=false

Guadeloupe / Saint Barthelemy and Saint Martin:

https://data.un.org/CountryProfile.aspx?crName=Guadeloupe
http://esa.un.org/wpp/Demographic-Profiles/pdfs/312.pdf

Martinique

http://www.prb.org/DataFinder/Topic/Rankings.aspx?ind=5

Puerto Rico / Cayman Islands/Anguilla/Sint Maarten

https://www.cia.gov/library/publications/the-world-factbook/rankorder/2091rank.html

Bonaire:

https://books.google.com/books?id=jdEgt78b0tIC&pg=PA465&lpg=PA465&dq=bonaire+infant+mortality+rate&source=bl&ots=4xOjl77rcY&sig=1EvCrl7YQ6U4v7hUwSfj6Ofew40&hl=en&sa=X&ei=smTkVNz3Nc-MoQTl3ICYBg&ved=0CDMQ6AEwAQ#v=onepage&q=bonaire%20infant%20mortality%20rate&f=false

Bahamas

https://www.cia.gov/library/publications/the-world-factbook/fields/2224.html

No data were included for the Falkland Islands (population around 2,000) , and Guantanamo (Cuba),

Gini Coefficient

Main Source: (University of Iowa)

http://myweb.uiowa.edu/fsolt/

Additional data downloaded from other sources:

OECD Income Distribution database (IDD):

http://stats.oecd.org/index.aspx?DatasetCode=CRSNEW

Bahamas

http://statistics.bahamas.gov.bs
http://www.paho.org/saludenlasamericas/index.php?option=com_docman&task=doc_view&gid=113&Itemid=

Cuba:

http://www.reuters.com/article/2008/04/10/us-cuba-reform-inequality-idUSN1033501920080410

Puerto Rico:

http://www.census.gov/prod/2011pubs/acsbr10-02.pdf

Suriname:

http://en.wikipedia.org/wiki/List_of_countries_by_income_equality

Trinidad and Tobago

http://www.tradingeconomics.com/trinidad-and-tobago/gini-index-wb-data.html (2012)
http://www.trinidadexpress.com/business-magazine/Allowing__govt_to_manage_better-197710671.html (1992)

Belize

http://planipolis.iiep.unesco.org/upload/Belize/Belize_MDG_2010.pdf
http://www.belizeinvest.org.bz/belize-trade-stats/

US Virgin Islands:

http://download.springer.com/static/pdf/301/art%253A10.1007%252FBF02717884.pdf?auth66=1424195957_ac4113337ac4ed57ad688109714166e4&ext=.pdf

Grenada:

http://www.gov.gd/egov/docs/reports/Grenada_CPA_Vol_1_Main_Report_Submitted.pdf

Aruba (N/A)

http://www.cbs.aw/terminology/179-about-terminology/g/588-gini-coefficient.html

Others:

https://books.google.com/books?id=Mt0gAwAAQBAJ&pg=PT415&lpg=PT415&dq=Aruba+GINI+coefficient&source=bl&ots=zBjDEjiiXl&sig=t5qquyhYbLainD1zQuPd5fUSJGE&hl=en&sa=X&ei=6IXjVPzvN4uLoQSFjYHICw&ved=0CB8Q6AEwATgK#v=onepage&q=Aruba%20GINI%20coefficient&f=false
